# Fungal Rutinosidase—Engineering of a Side Tunnel Steers Its Transglycosylation Potential

**DOI:** 10.1021/acs.jafc.6c02816

**Published:** 2026-05-18

**Authors:** Lucie Petrásková, Michael Kotik, Natalia Kulik, Andrea Vopálenská, Anna Šidáková, Katerina Brodsky, Vladimír Křen, Pavla Bojarová

**Affiliations:** a 86863 Institute of Microbiology of the Czech Academy of Sciences, Vídeňská 1083, Prague 4, CZ 142 00, Czech Republic; b Department of Organic Chemistry, Faculty of Science, Charles University, Albertov 6, Prague 2, CZ 128 00, Czech Republic; c Department of Analytical Chemistry, Faculty of Science, Charles University, Albertov 6, Prague 2, CZ 128 00, Czech Republic; ∥ Department of Health Care Disciplines and Population Protection, Faculty of Biomedical Engineering, Czech Technical University in Prague, nám. Sítná 3105, Kladno, CZ 272 01, Czech Republic

**Keywords:** active site, *Aspergillus niger*, *Aspergillus oryzae*, mutagenesis, rutinosidase, trans-glycosylation

## Abstract

Rutinosidases (α-l-rhamnosyl-β-d-glucosidases) are glycosidases (EC 3.2.1.168) that cleave the glycosidic bond between the aglycone and the disaccharide residue rutinose. Their dual substrate specificity is reflected in their activity toward both rutin (rutinosylated) and isoquercitrin (glucosylated) substrates. The structure of rutinosidase from *Aspergillus niger* (*An*Rut) features a side tunnel that influences the enzyme hydrolytic and transglycosylation activities. We present a mutagenesis study of this side tunnel, and the compartment forming the +1 binding subsite, resulting in seven variants with different active-site entry geometries. We show that the key side tunnel residues affect the catalytic and trans-rutinosylation potential of *An*Rut and compare these properties with rutinosidase from *Aspergillus oryzae*, which has a side groove instead of a side tunnel. The trans-rutinosylation abilities of the enzymes were tested using a diverse library of acceptors. This work expands the structure–function understanding of fungal rutinosidases and underlies the hypothesis that the engineering of the side tunnel can increase the transglycosylation-to-hydrolysis ratio.

## Graphical abstract

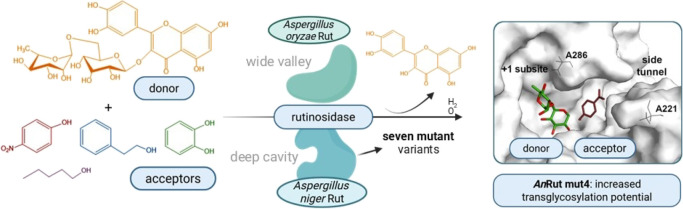

## Introduction

Rutinosidases (α-l-rhamnosyl-β-d-glucosidases) are a family of glycosidases (EC 3.2.1.168) that catalyze the cleavage of the glycosidic bond between the disaccharide rutinose and an aglycone. They belong to a subgroup of enzymes termed diglycosidases, classified into the GH5_23 and GH3 families.^1^ Rutinosidases typically cleave rutinose (α-l-Rha-(1→6)-β-d-Glc) from rutin (quercetin 3-*O*-rutinoside) or other rutinosides. They can also process isoquercitrin (quercetin 3-*O*-β-d-glucopyranoside) as an alternative substrate, affording quercetin and β-d-glucose.^2–4^ Rutinosidases are mainly found in plants and fungi.^1^ Their primary function in plants is the rapid hydrolysis of diglycosides, releasing toxic and/or signaling aglycones in a single step.^5,6^ This hydrolytic activity of rutinosidases is widely used in biotechnology to release aglycones of natural flavonoids for flavor and/or taste modification,^7^ typically in wine^8–10^ or tea.^11–13^ Another biotechnological application is the production of valuable commodities, rutinose and quercetin, from readily available rutin.^14^ Microorganisms use extracellular rutinosidases to break down plant-derived rutin, which serves as an energy and carbon source.^1,15^

All known rutinosidases utilize the retaining catalytic mechanism that produces rutinose in the β-anomeric configuration, identical to the parent substrate rutin.^1,2,16^ Therefore, rutinosidases should be able to perform glycosylation via kinetically controlled transglycosylation; however, only a few have been well studied in this respect, with transglycosylation demonstrated mainly in the GH5_23 family.^1^ When exploiting their dual catalytic activity, rutinosidases have been shown to transfer a rutinosyl moiety from rutin and a glucopyranosyl moiety from isoquercitrin to a wide range of acceptors, such as aromatic carboxylic acids, phenolic acids, inorganic azide, or primary and secondary alcohols.^3,4,17–20^ So far, only two structures of rutinosidases from the GH5_23 family have been resolved: those from *Aspergillus niger* (PDB 6I1A)^2^ and later from *Aspergillus oryzae* (PDB 6LA0).^21^ The structural analysis of rutinosidase from *A. niger* showed that the catalytic glutamates (Glu210 as the acid/base and Glu319 as the nucleophile) are located in a deep cavity and that the substrate access to the active site is further influenced by the side tunnel leading to the active site.^4^

This study aims to clarify the roles of amino acid residues that align the side (also called substrate or acceptor) tunnel, part of which forms the aglycone binding +1 subsite, in the hydrolysis and transglycosylation by *An*Rut. Our results show that mutagenesis in this region affects the catalytic properties and transglycosylation potential of *An*Rut as demonstrated by comparison with the structurally closely related rutinosidase from *Aspergillus oryzae* (*Ao*Rut), in which the side tunnel is substituted with a side groove. Additionally, using molecular modeling and molecular dynamics simulations, we show that mutations in this region can affect the binding of the substrate and the passage of the transglycosylation acceptor in *An*Rut by reshaping the side tunnel. The trans-rutinosylation properties of the two wild-type enzymes and four *An*Rut variants were investigated by using a library of structurally diverse acceptors including alcohols, polyphenols, flavonoids, and cholic acid derivatives. The present work brings new information about the underestimated synthetic potential of this hitherto poorly explored enzyme family.

## Materials and Methods

### Materials

*p*NP-rutinoside (4-nitrophenyl 6-*O*-(α-l-rhamnopyranosyl)-β-d-glucopyranoside; *p*NP-Rut; **5**) was prepared using a new synthetic procedure described in the Supporting Information, Section 1; the corresponding structural and spectral data are included. 4-Nitrophenyl β-d-glucopyranoside (*p*NP-Glc; **6**) was obtained from Lachema, Czech Republic.

Rutin (**7**) was obtained from Sigma-Aldrich, Germany, and isoquercitrin (**8**) was obtained by the bioconversion of rutin, as described previously.^22^ 2-Phenylethanol (**10**) was purchased from Fluka, catechol (**11**) was from Sigma, and *p*-nitrophenol (**12**) was from Lachema, Czech Republic. Quercetin (**13**), luteolin (**14**), myricetin (**15**), and taxifolin (**16**) were purchased from abcr (Karlsruhe, Germany), and cholic (**17**) and deoxycholic (**18**) acids were from Merck (Darmstadt, Germany). Pentanol (**9**) and all other chemicals and materials, unless stated otherwise, were purchased from VWR International (Stříbrná Skalice, Czech Republic) and were of analytical grade.

### Construction of Rutinosidase Variants

Site-directed mutagenesis of the variants *An*Rut mut1, mut3, mut4, and mut6 was performed using the QuikChange Lightning (Multi) Site-Directed Mutagenesis kit (Agilent Technologies, Santa Clara, California, USA) with *An*Rut wild type (WT)-encoding pPICZα A-based plasmid as a template.^2^ The mutagenic primers (Supporting Information, Table S2) were designed using the Primer Design program by Agilent Technologies. *An*Rut mut7 was constructed by replacing a 615 bp-long *An*Rut WT sequence with a 585 bp-long ds-DNA segment that contained the deletion. The synthesized DNA segment (GeneArt, Thermo Fisher Scientific) was restricted with *Sca*I and *Ssp*I and inserted into the *Sca*I/*Ssp*I-restricted and dephosphorylated *An*Rut-encoding pPICZα A-based plasmid. The resulting plasmid contained the modified *An*Rut-encoding gene with a deletion between Asn215 and Ala226. The *Ao*Rut and *An*Rut mut2 and mut5-encoding pPICZα A-based plasmids were obtained from Generay Biotech Co., Ltd. (Shanghai, China). The relevant DNA segments of the plasmid DNAs were verified by sequencing both DNA strands.

### Expression and Purification of Rutinosidases

The rutinosidase-encoding plasmids were linearized with *Sac*I and transformed into *Pichia pastoris* KM71H cells by electroporation. The resulting transformants were grown on YPDS agar plates containing 0.5–1.0 mg/mL of zeocin. Ten transformants were screened for high rutinosidase secretion into the culture medium. The best producer was selected for enzyme expression in two-stage liquid cultivations using BMGY and BMMY media with 0.5% methanol as an inducer, as previously described.^20^ The preparative rutinosidase production was performed in 0.2–0.8 L of BMMY culture medium. After cultivation, the biomass was removed by centrifugation, and the recombinant enzyme was purified from the supernatant by cation exchange chromatography using Fractogel EMD SO^3–^ resin (Merck Life Science s.r.o., Czech Republic) equilibrated with 10 mM sodium acetate buffer, pH 3.6. The bound proteins were eluted with a linear gradient of NaCl from 0 to 1.0 M.^20^ The *An*Rut variants mut3, mut4, and mut5 were obtained by ultrafiltration of the culture medium supernatant using Amicon Ultra-15 centrifugal filters (10 kDa cutoff; Merck Life Science s.r.o., Czech Republic).

### Enzyme Activity Assay

The enzyme activity with *p*NP-Glc and *p*NP-Rut substrates (2 mM) was determined spectrophotometrically at 420 nm in 50 mM McIlvaine buffer (pH 3.5) at 36 °C in a reaction volume of 50 μL. The reaction was stopped after 10 min by adding 1 mL of 0.1 M Na_2_CO_3_.^20^ One unit (U) of rutinosidase activity was defined as the amount of enzyme needed for the release of 1 μmol of *p*-nitrophenol per minute at pH 3.5 and 36 °C. Protein concentrations were determined according to the Bradford method with IgG as the calibration protein.^23^ The pH and temperature optima were determined with *p*NP-rutinoside by employing the standard enzyme activity assay. The pH optimum was determined in Britton–Robinson buffer pH 2.0–7.0 in 0.5 pH unit increments; The temperature optimum was determined in 5 °C increments over a range of 20 to 80 °C. Kinetic parameters were determined with *p*NP-rutinoside substrate in a concentration range of 6–150 mM under standard assay conditions. Due to the lack of a chromophore aglycone, the enzyme activities for rutin and isoquercitrin hydrolysis were determined by HPLC at 36 °C. Samples (20 μL; ≥5 time points per reaction) were withdrawn at 5 min intervals from a 300 μL reaction incubated at 36 °C under shaking (550 rpm) that contained 20 mM substrate, 10% v/v DMSO, and 50 μL of the appropriately diluted enzyme. A higher substrate concentration had to be used than for the spectrophotometric detection due to the lower sensitivity of the HPLC analysis. The addition of DMSO ensured full solubility of the substrates in the reaction mixture. The reactions ran for ca. 30 min and were terminated by heating (95 °C, 2 min). Before HPLC analysis, 100 μL of DMSO was added to the sample to completely dissolve all flavonoids, and the sample was centrifuged (13,094 × *g*, 2 min) and measured. For measurement conditions, see the section HPLC Analysis. The term “specific activity” as used in this work is defined as the amount (μmol) of the respective hydrolytic product formed from the given substrate (rutin, isoquercitrin, *p*NP-Rut, or *p*NP-Glc) under the catalysis by 1 mg of enzyme per minute. The conditions and substrate concentrations used for particular substrates are detailed in the Supporting Information.

### Analytical Transglycosylation Reactions

Rutin (**7**, 15.3 mg, 0.1 M) as a donor and the respective 0.3 M acceptor (pentanol (**9**) 8.2 μL, 2-phenylethanol (**10**) 9.3 μL, catechol (**11**) 8.3 mg, and *p*NP (**12**) 10.4 mg) were suspended/dispersed in McIlvaine buffer (50 mM, pH 3.5). The reported molarities represent nominal concentrations calculated from the total reaction volume (250 μL). The reaction was started by adding the respective enzyme (1 U/mL in the reaction mixture except for *An*Rut mut3 (0.5 U/mL), as it precipitates at higher concentrations) and ran for 120 h at a temperature of 36 °C and stirring at 550 rpm. The progress of the reaction was monitored by sampling (10 μL aliquots taken at 2, 4, 24, and 120 h). The reaction was stopped by thermal inactivation of the enzyme (99 °C for 2 min), centrifuged (13,094 × *g*, 2 min), and analyzed by HPLC. To quantify the ratio of transglycosylation/hydrolysis, the following calibration curves were generated using HPLC with evaporative light scattering detection (ELSD): for rutinose *A* = 109 247*c* – 88 891, *R* ^2^ = 0.9890, for 2-phenylethyl rutinoside *A* = 406 850*c* – 448 327, *R* ^2^ = 0.9925, for pentyl rutinoside *A* = 350 494*c* – 240 604, *R* ^2^ = 0.9905, for *p*NP-rutinoside *A* = 14 892c – 6518, *R* ^2^ = 0.9779, for catechol rutinoside *A* = 266 222*c* – 193 269, *R* ^2^ = 0.9875, where *A* is the peak area and *c* is the analyte concentration. The conditions of all HPLC analyses can be found in the following section, HPLC Analysis.

The reactions with quercetin (**13**; 22.7 mg; 0.3 M), luteolin (**14**; 21.5 mg; 0.3 M), myricetin (**15**; 24 mg, 0.3 M), taxifolin (**16**; 22.8 mg; 0.3 M), cholic (**17**; 30.6 mg; 0.3 M), or deoxycholic (**18**; 29.4 mg; 0.3 M) acids as acceptors and 0.1 M rutin (**7**) as a donor were performed in McIlvaine buffer (50 mM, pH 3.6) at a temperature of 36 °C and stirring at 550 rpm. The total volume of the reaction was 250 μL. The reaction took place in suspension because the acceptors are only partially soluble in water. The reaction was started by adding the respective enzyme (2 U/mL in the reaction mixture) and ran for 120 h. The progress of the reaction was monitored by sampling: 20 μL aliquots of supernatant were taken at 2, 3, 4, 24 h, and 20 μL aliquots of suspension were taken at 48, 72, and 120 h and 40 μL of DMSO was added before HPLC analysis. The reaction was stopped by thermal inactivation of the enzyme (99 °C for 2 min).

### HPLC Analysis

HPLC analyses were performed on a Shimadzu Prominence LC analytical system consisting of a LC-20AD binary HPLC pump, a DGU-20A_3_ degasser, an SIL-20ACHT cooling autosampler, a CTO-10AS column oven, a CBM-20A system controller, and an SPD-20MA diode array detector (Shimadzu, JP). Reaction mixtures with rutin (**7**) or isoquercitrin (**8**) substrates were diluted in 100 μL of DMSO and analyzed on a Chromolith RP-18e column (100 × 3 mm, Merck, Germany) equipped with a Chromolith RP-18e guard column (5 × 4.6 mm). The binary gradient elution was as follows: mobile phase A = 5% acetonitrile, 0.1% formic acid, mobile phase B = 80% acetonitrile, 0.1% formic acid; the gradient for measuring enzyme activity was as follows: 0–3 min 7–30% B, 3–5 min 30% B, 5–7 min 30–7% B, 7–7.5 min 7% B to equilibrate the column; flow rate 1.5 mL/min, 25 °C; injection volume 1 μL. The transglycosylation activity of the enzymes with pentanol (**9**), 2-phenylethanol (**10**), catechol (**11**), quercetin (**13**), luteolin (**14**), myricetin (**15**), and taxifolin (**16**) as acceptors was analyzed on a Luna NH_2_ column (5 μm, 150 × 4.6 mm, 100 Å) equipped with a NH_2_ security guard column (4 × 3 mm) (both Phenomenex, California, USA). Binary gradient elution was as follows: mobile phase A = acetonitrile, mobile phase B = water; gradient for measurement was as follows: 0–8 min 21–30% B, 8–12 min 30–50% B, 12–13 min 50% B, 13–15 min 21% B to equilibrate the column; flow rate 1.1 mL/min, 25 °C; injection volume 1 μL. The transglycosylation activity of the enzymes with *p*NP (**12**), cholic (**17**), or deoxycholic (**18**) acid as acceptors was performed on a Chromolith column with mobile phases as mentioned above, binary gradient 0–2 min 0% B, 2–7 min 0–90% B, 7–8 min 90% B, 8–11 min 90–0% B, 11–14 min 0% B to equilibrate the column; flow rate 1.2 mL/min, 25 °C; injection volume 1 μL.

The photodiode array (PDA) detector was set from 200 to 400 nm, and the wavelength at the substrate absorption maximum was extracted. For the evaporative light scattering detector (ELSD), the set parameters were as follows: nebulization temperature 40 °C, evaporation temperature 85 °C, and flow rate of gas (N_2_) 0.8 mL/min.

MS detection was performed by MS-ESI; the parameters were as follows: positive and negative mode; ESI interface voltage, 4.5 kV, −3.5 kV; detector voltage, 1.15 kV; nebulizing gas flow, 1.5 L/min; drying gas flow, 15 L/min; heat block temperature, 200 °C; the temperature of desolvation line pipe, 250 °C, SCAN mode 200–500 *m*/*z*; all chromatograms were analyzed using software LabSolutions ver. 5.75 SP2 (Shimadzu, Kyoto, Japan).

### Molecular Dynamics Simulations and Docking

For structural analysis, we used the respective crystal structures of *An*Rut (6I1A)^2^ and *Ao*Rut (6LA0)^21^ WT enzymes. Structures of rutin and isoquercitrin were downloaded from the PubChem database (https://pubchem.ncbi.nlm.nih.gov/). Other ligand and structure modifications were done in YASARA (version 24.4.10_S).^24^ Mutant structures were built and optimized in YASARA based on the *An*Rut WT crystal structure, followed by short energy minimization using the standard protocol.^24^ In variants mut2–mut6, simple substitutions with smaller residues (Ala, Gly) or a single-residue deletion were introduced. Previous experimental studies have shown that such mutations usually induce only local structural perturbations without altering the global fold.^25,26^ Therefore, we consider the use of the *An*Rut crystal structure (6I1A)^2^ as a template for modeling these variants to be justified. For the mut1 variant, two rotamers of residue *I*222 were modeled to account for possible local conformational variability. For mut7, more pronounced structural effects could be expected; therefore, additional equilibration and molecular dynamics simulations were performed to assess structural stability. To optimize the modeled variants, all structures were equilibrated. As the first step of minimization, only the mutated residues and their neighboring residues were minimized whereas the rest of the protein was kept fixed. Then, the residues within 5 Å of the mutated residues were released for minimization *in vacuo* using the NOVA force field. Finally, the complete structure was minimized in both vacuum and water. Docking of ligands was done in AutoDock4 using the Lamarckian Genetic Algorithm,^27^ and the pose selection was guided by previously published data.^2^ Initial ligand orientation was corrected by energy minimization in YASARA.

Molecular dynamics (MD) simulations were run in YASARA with the AMBER15IPQ force field in TIP3P water, with the number of ions required only for system neutralization. Ligand parameters were derived by implementing the AutoSMILES algorithm; carbohydrate parameters were taken from the GLYCAM06 force field.^28^ Missing parameters were derived from GAFF2.^29^ All MD simulations were conducted for 100 ns in the NPT ensemble (pressure was set up to keep the water density at 0.997 g/mL, *T* = 300 K) with recalculation of intramolecular forces every 2 fs and intermolecular forces every 1 fs.

Final analysis of interactions was done with a YASARA tool, GetContacts (https://getcontacts.github.io/). Free energy calculation was done for the 10 selected snapshots from the last 20 ns of MD simulation by AutoDock Vina (except for ligands with an abrupt increase in root mean square deviation (RMSD) after 80 ns; in those cases, we used a 60–80 ns time window).^30,31^ We assumed that the side tunnel is formed if the common surface between loops aa 214–225 and aa 284–291 in *An*Rut WT or between loops aa 194–205 and aa 264–270 in *Ao*Rut WT was not zero. The data were analyzed over a period of 0–100 ns of MD simulation. If the common surface was equal to zero, the tunnel was assigned as a groove. To calculate the percentage of changes in the side tunnel, the data from all snapshots were divided by the number of snapshots (1000).

To analyze the passage of the transglycosylation acceptor through the side tunnel or groove, we used structures from MD simulations of *Ao*Rut WT, *An*Rut WT, or its mutant variants with the intermediate structure of rutinose covalently bound to E319 in *An*Rut or to E298 in *Ao*Rut, taken from 80–100 ns of MD (400 snapshots). The passage calculations were performed in Caver Analyst 2.0;^32,33^ the starting point was the expected position of the transglycosylation acceptor oxygen before product formation (between carboxyls of E210, E319, and hydroxyl of Y284 in *An*Rut; or carboxyls E191, E298, and hydroxyl of Y264 in *Ao*Rut). A minimum probe radius of 1.5 Å, a shell radius of 3 Å, and a depth of 4 Å were used as calculation parameters. The snapshot with the narrowest passage identified by Caver Analyst 2.0 was further used for modeling the acceptor trajectory in the side tunnel and for energy calculations with CaverDock 1.2.^34,35^ For calculating the free energy of binding, we implemented the AutoDock Vina 1.1.2 algorithm.^30,31^ Docking results were analyzed from two perspectives: (*i*) correctness of the binding pose and (*ii*) favorable energy for entering the active site from the side tunnel/groove. The transglycosylation acceptor was favorably (correctly) positioned for the glycosylation step if its hydroxyl group was located between the carbonyl of the catalytic glutamic residue and C-1 of the rutinoside intermediate (within 4 Å).

Surface calculations were performed and visualized using ChimeraX,^36^ YASARA, and PyMOL.

## Results

### Synthesis of *p*NP-Rutinoside Substrate

*p*-Nitrophenyl rutinoside (4-nitrophenyl 6-*O*-(α-l-rhamnopyranosyl)-β-d-glucopyranoside; *p*NP-Rut; **5**) is an essential analytical substrate for enzymatic reactions with rutinosidases. Due to its prohibitive price (*e.g*., Biosynth Ltd., UK, 217 $ per 1 mg), this compound is practically inaccessible for routine activity testing on a larger scale. To overcome this hurdle, we developed an original four-step synthesis starting from rutinose (**1**; Scheme 1). Rutinose was obtained on a preparative scale by selective hydrolysis of rutin catalyzed by recombinant *An*Rut WT, as published previously.^14^ The first step of our synthesis was peracetylation of rutinose (**1**) using acetic anhydride/pyridine to form peracetate **2** (80% yield). Subsequent bromination of peracetylated rutinose **2** afforded glycosyl α-bromide **3**, which was directly subjected to Koenigs–Knorr glycosylation catalyzed by silver carbonate in the presence of 4-nitrophenol to afford glycoside **4** (42% yield over two steps). The final Zemplén deacetylation of glycoside **4** yielded *p*-nitrophenyl rutinoside (**5**; 72%), which was fully characterized by ^1^H and ^13^C NMR, HPLC, and HRMS (Supporting Information, Table S1 and Figures S1–S4). For further details of the synthetic procedure, see Supporting Information, Section 1.

**Scheme 1:**
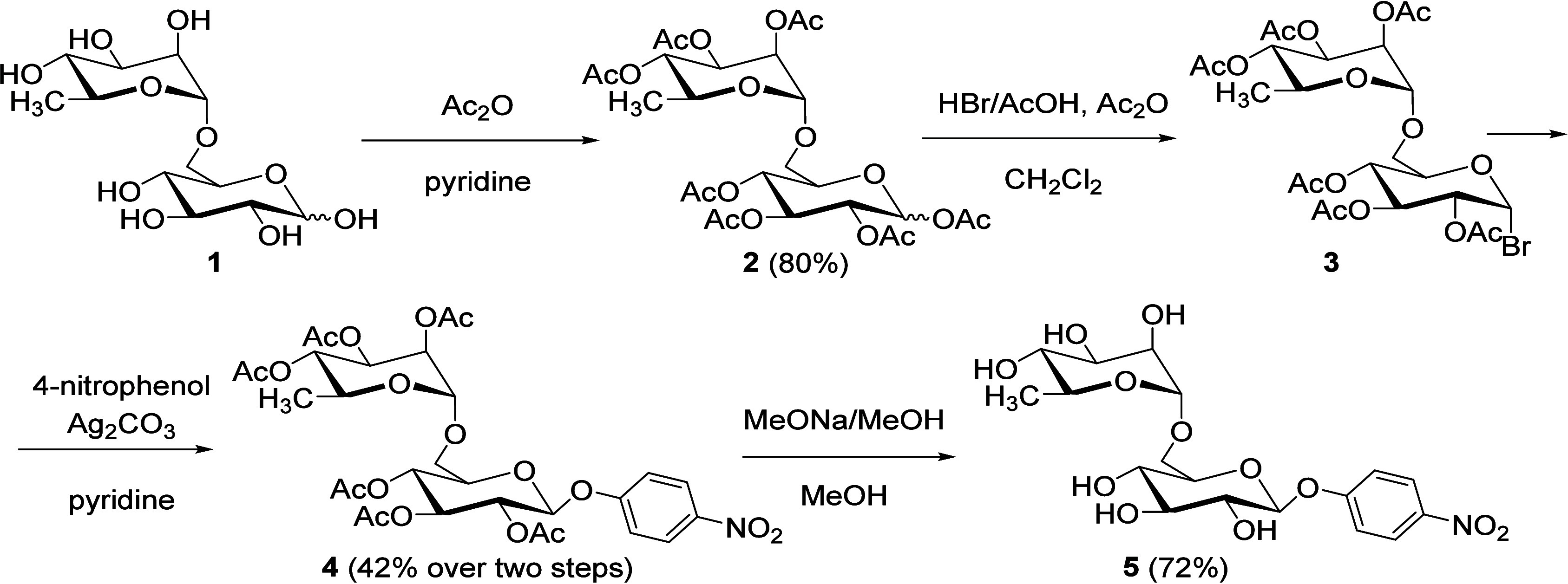
Synthesis of 4-Nitrophenyl 6-*O*-(α-l-Rhamnopyranosyl)-β-d-glucopyranoside (**5**) from Rutinose (**1**)

### Design and Production of *An*Rut Mutant Variants

Based on a comparison between the crystallographic structures of the tunnel-containing *An*Rut^2^ and the structurally very similar, albeit tunnel-lacking *Ao*Rut (with 70% sequence identity),^21^ we designed a series of genetic modifications intended to either widen or spatially restrict/close the side tunnel in *An*Rut. The aim was to gain deeper insight into the role of the side tunnel and its amino acid residues in substrate hydrolysis and transglycosylation reactions. These considerations led to the design of the *An*Rut variants mut1, mut2, and mut5–mut7. Furthermore, we hypothesized that replacing one or two aromatic residues (Phe and Tyr) located in the loop covering the aglycone binding site with a smaller hydrophobic residue, such as Ala, would open the active site for bulkier acceptors, thereby improving their access to the glycosyl-enzyme intermediate and allowing them to compete with water access. As suggested in the literature, an important aspect of strong enzymatic transglycosylation abilities is the reduced accessibility of water to the anomeric carbon of the glycosyl-enzyme intermediate in the active site.^37^ This hypothesis led to the design of the *An*Rut variants mut3 and mut4. The list of all prepared variants and two reference WT rutinosidases, including the changes in their tunnel structures imposed by their respective mutations during MD simulation of substrate-free mutant variants, is shown in Table 1.

**Table 1:** List of Enzymes Used in This Study

Enzyme variant^a^	Genetic modification	Description of the changes in side tunnel^b^
***An*** **Rut WT**	none	dynamic side tunnel transforms into a groove in 50% MD^c^
***Ao*** **Rut WT**	none	side groove in 96% MD^c^
*An*Rut mut1	*An*RutG222I	narrow, rather hydrophobic side tunnel in 96% MD^c^
*An*Rut mut2	*An*RutG222–/T223A/F261G/Y284A/K307A	side tunnel with unstable width in 91% MD^c^
***An*** **Rut mut3**	*An*RutF221A	side tunnel broader than WT in 82% MD^c^
***An*** **Rut mut4**	*An*RutF221A/Y286A	side groove in 84% MD^c^
***An*** **Rut mut5**	*An*RutH288–/E287G/M289A	neutrally charged side groove in 95% MD^c^
***An*** **Rut mut6**	*An*RutM218G/V219G/H288G	negatively charged side tunnel in 95% MD^c^
*An*Rut mut7	*An*RutΔ(^216^TNMVVFGTPL^225^)	solvent-accessible side groove in 100% MD^c^

a The enzyme variants in **bold** were used for further characterization and transglycosylation reactions.

b For tunnel definition, see Figure 3 in ref 4 for tunnel structures in individual variants; see Figure 1.

c The calculation of the side tunnel/groove conformation is described in detail in the Materials and Methods. The percentage [%] refers to MD simulation time (100% means all MD simulation time, *i.e*., 100 ns).

For representative structures of substrate-free enzyme variants showing the most frequently observed conformation of the side tunnel/groove; see Figure 1.

**Figure 1:**
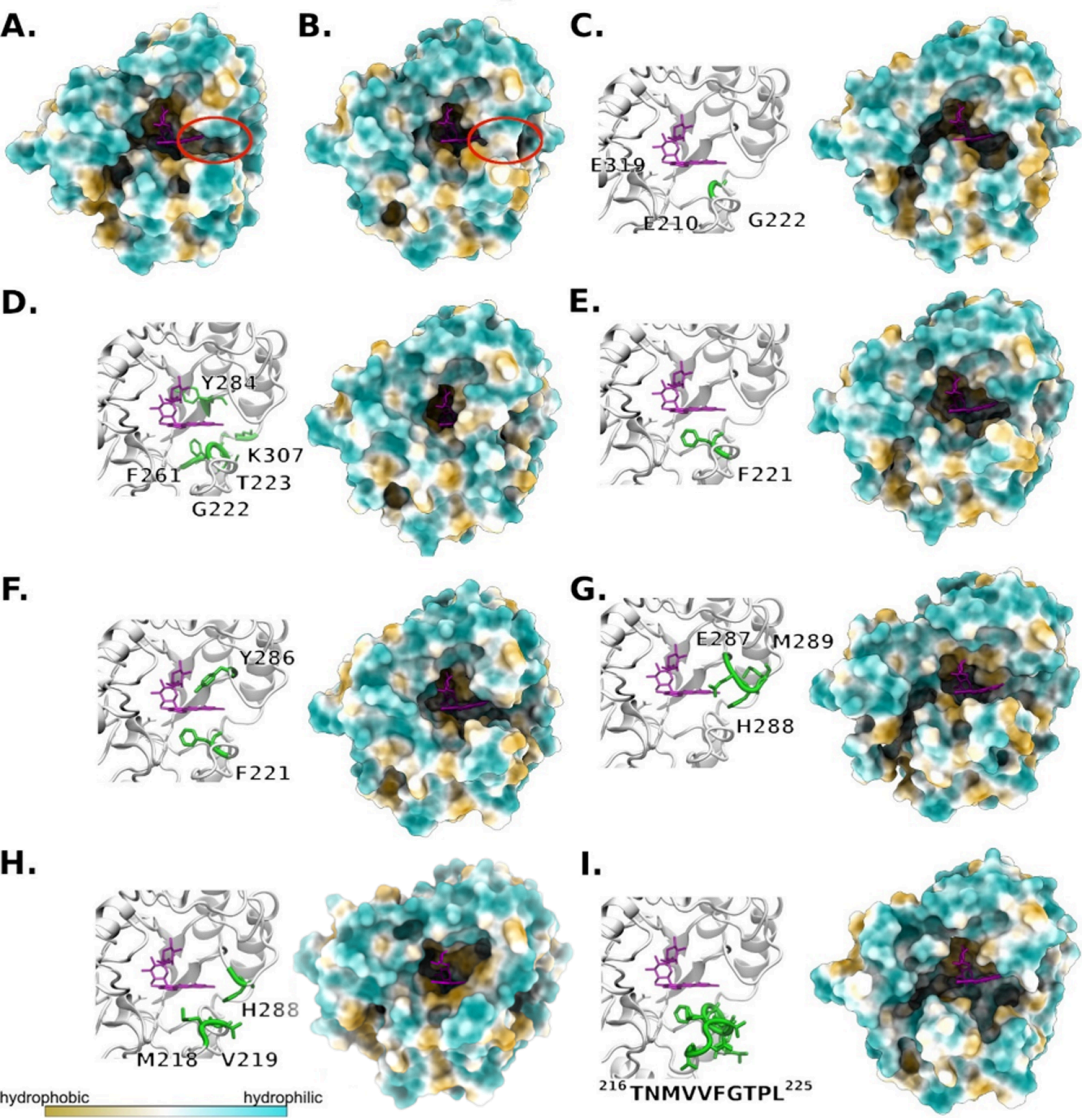
Enzyme structures after 100 ns of MD simulation in the absence of substrate viewed from the top of the active site entrance. The rutin position is interpolated from *An*Rut WT to determine the active site. In mutant variants, the left panel maps mutated residues (green) in the *An*Rut WT structure (cartoon representation); catalytic residues are shown as gray sticks; they are labeled only in (C). The right panel shows the hydrophobic surface of the studied enzymes after 100 ns of MD simulation, calculated with ChimeraX. (A) *Ao*Rut with a side groove (red circle); (B) *An*Rut with a formed side tunnel (red circle); (C) mut1; (D) mut2; (E) mut3; (F) mut4; (G) mut5; (H) mut6; (I) mut7.

The rutinosidase-encoding genes were cloned downstream of the α-factor signal sequence of the pPICZα A vector in the constructed plasmids, enabling the secretion of the recombinant proteins into the culture medium. The plasmids for the expression of *An*Rut mut2 and mut5 were obtained commercially, while those for the expression of *An*Rut mut1, mut3, mut4, and mut6 were prepared by site-directed mutagenesis (Supporting Information, Table S2). *An*Rut mut7 was prepared by deleting a 10-amino-acid-encoding segment between N215 and A226. All variants were heterologously expressed in *P. pastoris* as detailed in the Materials and Methods. Notably, despite repeated attempts, no protein expression of *An*Rut mut1 or mut2 was detected, neither in the culture medium nor intracellularly as expected.

The WT enzymes, as well as the five remaining *An*Rut variants mut3–mut7 (in contrast to the mut1 and mut2 variants, which were apparently unstable and have never been observed by SDS-PAGE in the medium), were then successfully produced in preparative amounts and purified by cation exchange chromatography. The purification yields are summarized in the Supporting Information, Table S3. *An*Rut mut7 was produced in a very small quantity and had virtually no detectable activity in the culture medium. Therefore, it was not selected for further biochemical characterization and transglycosylation reactions. In contrast, mut5 and mut6 variants, along with the WT enzymes, were sufficiently active in the culture medium with *p*NP-Rut. The mut3 and mut4 variants showed no detectable activity in the culture medium with *p*NP-Rut as a substrate; however, the specific activities were detectable (≤0.1 U/mg) after ultrafiltration. Purification of the mut3, mut4, and mut5 variants by ion exchange chromatography resulted in their substantial inactivation, even after optimization and testing of different pH values and buffers. Therefore, given the high selectivity of our heterologous expression system and the absence of other contaminating proteins in the culture medium, we decided to use these variants for further experiments only after an ultrafiltration step without a column chromatography purification step. The enzyme purity of the produced enzyme variants was analyzed by SDS-PAGE, as shown in the Supporting Information, Figure S5.

### *In Silico* Analysis of the Impact of Introduced Mutations on the Structure of *An*Rut Mutant Variants

Through molecular dynamics (MD) simulations of the structures of the substrate-free mutant variants, we examined qualitative structural trends associated with the introduced mutations and used these analyses to support hypotheses regarding the effects of these mutations on enzyme stability and active-site architecture, primarily using parameters such as RMSD (root mean square deviation; Supporting Information, Figure S10), RMSF (root mean square fluctuation; Supporting Information, Figure S11), and structural alignment. *An*Rut mut1 and mut7 showed the largest structural changes compared with the WT enzyme, with unstable RMSD during 100 ns of MD (Supporting Information, Figure S10A). In contrast, variants mut3–mut6 reached equilibrium after 70 ns (Supporting Information, Figure S10B), with mut4 being the most stable structure of the mutant series.

To assess the protein flexibility, RMSF was analyzed. Mut1 and mut2 exhibited very high flexibility in the region of aa 216–225, which contains the mutations (Supporting Information, Figure S11). The G222I substitution in mut1 could not be stabilized by the formation of a hydrophobic patch,^38^ because no Ile, Val, or Leu was found in the vicinity of the residue. The structural alignment of the substrate-free mutant structures and *An*Rut WT with docked rutin after 100 ns of MD was used to verify the accessibility of the active site to substrates and the ability to form functional interactions. In mut2, the catalytic nucleophile E319 rotated during MD and shifted to a position freed by the Y284A mutation (Supporting Information, Figure S12A). Deletion of the aa 216–225 loop resulted in a more water-exposed binding site with a very high flexibility in mut7 (Supporting Information, Figure S11A,B).

### Substrate Specificity and Biochemical Characterization of *An*Rut Mutant Variants

For the analysis of substrate binding, the RMSDs of the substrate in the enzyme active site, hydrogen bonding, hydrophobic interactions, and free energy of substrate binding were calculated. Structural alignment was used to compare the spatial orientation of the substrate with respect to that of the WT enzyme and the natural substrate (rutin).

When *p*NP-Rut (**5**) was used as a substrate, the specific activities of the mutant enzyme variants were significantly lower than those of *An*Rut WT (1.4 U/mg), especially for mut3 and mut4. In contrast, mut5 and mut6 showed slightly higher specific activities, with a less dramatic decrease in specific activity of 57 and 81%, respectively, compared with *An*Rut WT (1.4 U/mg). The highest specific activity with **5**, 4.0 U/mg, was found for the other enzyme, WT enzyme *Ao*Rut (Table 2). Since rutinosidases are known for their dual substrate specificity, the β-glucosidase activity was also investigated using *p*NP-Glc (**6**). There is a substantial difference between *An*Rut and *Ao*Rut WTs: While *Ao*Rut is a distinct rutinosidase with over 50-fold weaker glucosidase activity, *An*Rut is much less selective, with only 4-fold stronger rutinosidase activity. None of the mutations introduced in mut3–mut6 altered the prevalence of rutinosidase activity over glucosidase found in the WT, though their ratio differed considerably in the series (Rut/Glc = ca. 1.4–15). Interestingly, when natural substrates of rutinosidase—rutin (**7**; Rut) vs. isoquercitrin (**8**; Glc)—were examined, the selectivity was much less pronounced. While *Ao*Rut maintained prevalent rutinosidase activity (over 3-fold), *An*Rut WT predominantly behaved as a glucopyranosidase (Rut/Glc = 0.5), as did all its mutants tested (Rut/Glc = 0.3–0.9). This different behavior underlines the pivotal role of the flavonoid aglycone, which provides the majority of substrate interactions in the active site.

**Table 2:** Specific Activities of Rutinosidase Variants with Various Substrates

Variant^a^	Specific activity [U/mg]			
	*p*NP-Rut (**5**)	*p*NP-Glc (**6**)	Rutin (**7**)	Isoquercitrin (**8**)
*Ao*Rut WT	4.0 ± 0.3	0.077 ± 0.001	8.17 ± 0.31	2.38 ± 0.02
*An*Rut WT	1.47 ± 0.03	0.353 ± 0.003	3.32 ± 0.18	6.4 ± 0.3
*An*Rut mut3	0.020 ± 0.001	0.038 ± 0.001	0.95 ± 0.02	3.7 ± 0.1
*An*Rut mut4	0.098 ± 0.003	0.069 ± 0.002	0.75 ± 0.03	0.98 ± 0.02
*An*Rut mut5	0.70 ± 0.03	0.042 ± 0.002	1.398 ± 0.008	1.89 ± 0.05
*An*Rut mut6	0.35 ± 0.05	0.103 ± 0.005	1.40 ± 0.02	1.55 ± 0.04

a *An*Rut mut1 and *An*Rut mut2 were not expressed; the amounts and activity of mut7 were insufficient for the screening. The term “specific activity” as used in this work is defined as the amount (μmol) of the respective hydrolytic product formed from the given substrate (rutin, isoquercitrin, *p*NP-Rut, or *p*NP-Glc) under the catalysis by 1 mg of enzyme per minute. The conditions and substrate concentrations used for particular substrates are detailed in this Section (colorimetric determination for *p*NP-Rut and *p*NP-Glc; HPLC determination for rutin and isoquercitrin).

*p*-Nitrophenyl rutinoside (*p*NP-Rut; **5**) is a colorimetric substrate that enabled a straightforward determination of biochemical (Table 3) and kinetic parameters (Table 4 and Supporting Information, Figure S7). To compare the biochemical parameters of the prepared mutants (*An*Rut mut3–mut6) with the parent WT enzyme, we determined their pH and temperature optima (Table 3). None of the mutations had a significant effect on the temperature or pH optima. Only minor differences were observed, particularly in the temperature dependence profile (Supporting Information, Figure S6). The pH optima ranged from 2.5 to 3.0, and the temperature optima ranged from 45 to 55 °C. For convenience, these characterizations were performed using a spectrophotometric assay with *p*NP-Rut (**5**) instead of the lengthy and material-consuming HPLC-based activity determination used for the native substrates **7** and **8.**

**Table 3:** pH and Temperature Optima of Rutinosidase Variants Assessed with *p*NP-Rut Substrate (**5**)­^a^

Variant	pH Optimum	Temperature optimum [°C]
*An*Rut WT^b^	3	50
*An*Rut mut3	2.5	55
*An*Rut mut4	2.5	45
*An*Rut mut5	3	50
*An*Rut mut6	2.5	55

a *An*Rut mut1 was not expressed; *An*Rut mut2 and mut7 did not have sufficient activity and amount for the screening.

b Data adopted from ref 20.

**Table 4:** Kinetic Parameters of Rutinosidase Variants Assessed with *p*NP-Rut Substrate (**5**)­^a^

Variant	*K*_M_ [mmol/L]	*k*_cat_ [s^–1^]	*k*_cat_/*K*_M_ [L/mmol/s]
*Ao*Rut WT	7.9 ± 0.7	10.4 ± 0.3	1.32
*An*Rut WT	51 ± 4	32 ± 1	0.62
*An*Rut mut3	89 ± 7	3.1 ± 0.1	0.03
*An*Rut mut4	61 ± 5	3.0 ± 0.1	0.05
*An*Rut mut5	29 ± 3	5.8 ± 0.2	0.2
*An*Rut mut6	30 ± 3	19.7 ± 0.7	0.65

a *An*Rut mut1 and *An*Rut mut2 were not expressed; mut7 did not have sufficient activity and an amount for the screening.

The determination of kinetic parameters with the colorimetric substrate *p*NP-Rut (Table 4 and Supporting Information, Figure S7) revealed that mutagenesis near the side tunnel or the +1 binding subsite had little effect on the affinity to this substrate. While *An*Rut WT had a *K*_M_ of 51 mM, the *K*_M_ values of its mutant variants ranged from 29 to 90 mM. Even converting the side tunnel to a groove (mut4, mut5, and mut6) did not bring this value closer to that of *Ao*Rut WT, which exhibited a more than 6-fold stronger affinity to *p*NP-Rut, with a *K*_M_ in the single-digit millimolar range. An even clearer difference appeared in the turnover number (*k*_cat_). Except for mut6, the mutant variants showed a strong decline in *k*_cat_ compared with the two WTs, ca 5- to 10-fold lower than the parent enzyme (32 s^–1^). This strong decrease was also reflected in the reduced catalytic efficiencies of the mutant variants. However, this is not surprising, as this feature is found in most mutant variants^39,40^ and does not necessarily negatively affect synthetic capabilities, as explained below. In this respect, *An*Rut mut6 is exceptional, since its catalytic efficiency matches that of the WT relatively well.

### *In Silico* Analysis of the Substrate Specificity of *An*Rut Mutant Variants

The enzyme affinity to the substrates was estimated by the free energy of binding (Supporting Information, Figure S13). The natural substrate rutin (**7**) strongly interacted with both *Ao*Rut WT and *An*Rut WT, mainly via its aglycone quercetin. The average number of hydrogen bonds with the quercetin aglycone during MD simulation was 3.13 for *An*Rut and 2.14 for *Ao*Rut (Supporting Information, Table S5). The residues aa 218–222 and aa 284–288 in *An*Rut, or aa 198–201 and aa 264–267 in *Ao*Rut, located in the side tunnel/groove, contributed to the interaction with rutin. The loops above the aglycone binding site restricted quercetin mobility (lower RMSD; Supporting Information, Figure S14A) and decreased hydration in the active site. The number of water molecules forming the first hydration shell (within ca. 0.2 nm) was significantly smaller in *An*Rut WT, mut3, and mut4. The number of water molecules within 0.3 nm of rutin bound in the enzyme active site was almost 50% lower than for unbound rutin in water (Supporting Information, Figure S15).

The mutation of two aromatic residues in the aglycone binding site (+1 subsite) of the side tunnel in *An*Rut, F221A and Y286A, decreased the free energy of binding for rutin as shown in mut4 (Supporting Information, Figure S13), and reduced the number of hydrogen bonds and π-stacking interactions (Supporting Information, Figure S16A,B), resulting in a significant reorientation of the quercetin aglycone (Supporting Information, Figure S17). The H288 mutation in both mut5 and mut6 increased solvation of the quercetin aglycone compared with *An*Rut WT (Supporting Information, Figure S15), lowered the number of hydrogen bonds (Supporting Information, Figure S16), and decreased the free energy of binding (more pronounced in mut6; Supporting Information, Figure S13). Without substrate, mut6 formed a tunnel during 95% of the MD simulation time; however, upon binding rutin, it changed geometry to a groove throughout the MD simulation.

In contrast to rutin, isoquercitrin (**8**) is glycosylated with a single glucosyl moiety. This modification did not impair the strong interaction of the quercetin aglycone in the active site, particularly in *Ao*Rut WT (with the E31 residue). *Ao*Rut WT formed more interactions than *An*Rut WT with residues close to the side tunnel (E287, H288 in *An*Rut WT and E267 in *Ao*Rut WT—see Supporting Information, Figure S18A–C). Isoquercitrin could not be stabilized in the active site of either mut3 or mut4 due to the increased RMSD (Supporting Information, Figures S14B and S18D,E). Mut4 bound isoquercitrin rather poorly (Supporting Information, Figure S13) since the quercetin aglycone shifted into the widened side tunnel, further from catalytic E210, forming new hydrogen bonds with the backbone of Y284 (Supporting Information, Figure S18E).

*p*-Nitrophenyl rutinoside (*p*NP-Rut; **5**) was not stabilized in *An*Rut mut3 and mut4 according to MD simulations, as indicated by the unstable and increasing RMSD (Supporting Information, Figure S14). During the MD simulation, the *p*NP group occasionally occupied the site of the mutated F221 in mut3, increasing the distance between the substrate and the catalytic E210, which could potentially hinder hydrolysis (Figure S19A). In mut4, many hydrogen bonds with *p*NP-Rut were lost during MD because the *p*NP aglycone shifted to the position of the mutated Y286 and formed a stacking interaction with Y284, leading to displacement of *p*NP-Rut in the active site (Supporting Information, Figure S19B). In mut6, the orientation of *p*NP-Rut was similar to that of WT, with a low RMSD and a comparable number of hydrogen bonds (Supporting Information, Figures S14 and S16).

*p*-Nitrophenyl β-d-glucopyranoside (*p*NP-Glc; **6**) behaved differently in *An*Rut WT and *Ao*Rut WT during MD. In *An*Rut WT, the *p*NP aglycone remained oriented toward the loops framing the side tunnel and could form hydrogen bonds with backbone atoms of Y286 and E287. In contrast, in *Ao*Rut WT, the *p*NP aglycone moved further from its side groove (Supporting Information, Figure S20A–D), as indicated by the increased RMSD (Supporting Information, Figure S14D). *p*NP-Glc also formed more hydrogen bonds and stacking interactions with *An*Rut WT than with *Ao*Rut WT (Supporting Information, Figure S16). In all mutants and in *Ao*Rut, *p*NP-Glc remained relatively distant from the catalytic residue (E210 in mutants and E191 in *Ao*Rut, Supporting Information, Figure S20E), making hydrolysis more difficult.

### Transglycosylation Potential of *An*Rut Mutant Variants

The trans-rutinosylation potential of the mutant and WT enzymes was assessed in a series of analytical reactions, in which rutin served as a donor and pentanol (**9**), phenylethanol (**10**), catechol (**11**), or *p*-nitrophenol (**12**) served as an acceptor (Figure 2 and Supporting Information, Table S4). Transglycosylation activity was observed for all of the enzymes tested. As a representative parameter of transglycosylation potency, we calculated the molar ratio of the respective transglycosylation product to the hydrolytic product rutinose at the same reaction times (2, 3, 24, and 120 h). This ratio can be interpreted as the ratio of transglycosylation (TG) to hydrolytic (H) activities in the reaction and was denoted as the transglycosylation/hydrolysis ratio (TG/H). The molar concentrations of all compounds were determined by calibration with previously prepared authentic standards.^20,4^

**Figure 2:**
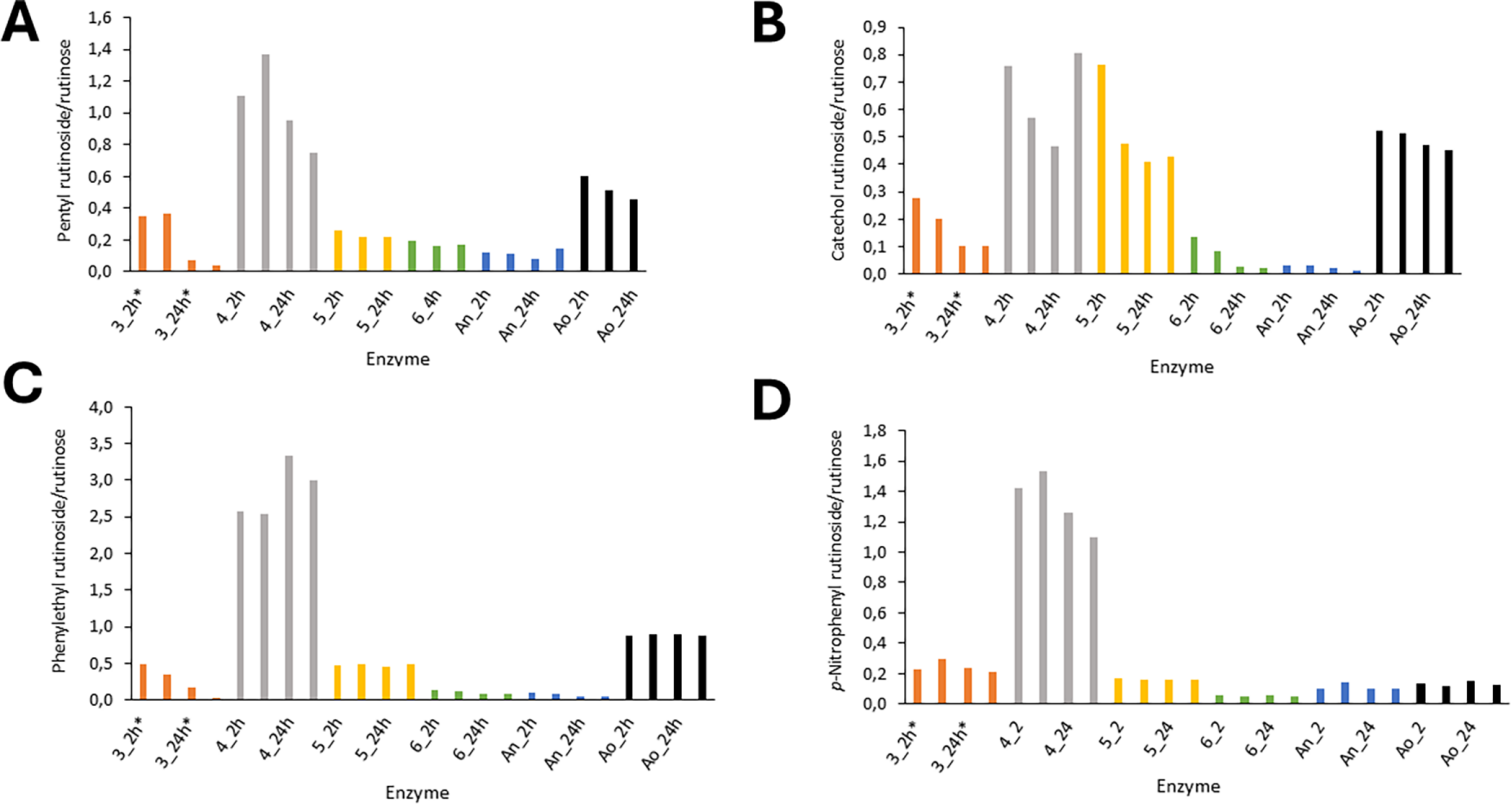
Comparison of the transglycosylation behavior of mutants and WT enzymes with rutin as a donor and pentanol (A), catechol (B), phenyl ethanol (C), and *p*-nitrophenol (D) as acceptors. The *x*-axis shows the numbers of respective mutant variants (orange for *An*Rut mut3, gray for *An*Rut mut4, yellow for *An*Rut mut5, green for *An*Rut mut6, blue for *An*Rut WT, and black for *Ao*Rut WT) at different times (2, 3, 24, and 120 h). The *y*-axis shows the molar ratios of the respective transglycosylation product and rutinose calculated from the calibration curves. The calibration equation for each substance was as follows: *A* = 350494 *c* – 240604, *R* ^2^ = 0.9905 for pentanol, *A* = 266222 *c* – 193269, *R* ^2^ = 0.9875 for catechol, *A* = 406850 *c* – 448327, *R* ^2^ = 0.9925 for phenyl ethanol, *A* = 14892 *c* – 6518, *R* ^2^ = 0.9779 for *p*-nitrophenol, and *A* = 109247 *c* – 88891, *R* ^2^ = 0.9890 for rutinose. *The concentration of *An*Rut mut3 in the transglycosylation reaction was 0.125 U (0.5 U/mL), as it otherwise precipitated at higher concentrations; the concentration of enzymes mut4, mut5, mut6, and the WT enzymes was 0.25 U in the transglycosylation reaction (1 U/mL).

When comparing the transglycosylation potential of both benchmark WT enzymes (Figure 2 and Supporting Information, Table S4), the trans-rutinosylation capability of *An*Rut WT never exceeded a TG/H ratio of 0.2 with any acceptor, which made it the least efficient transglycosylation tool in the series. *Ao*Rut behaved as a substantially better transglycosylation tool than did *An*Rut. It achieved TG/H ratios of 0.5–0.6 with its best acceptors, pentanol and catechol, which ranked it among the well-performing transrutinosylating variants. Notably, *An*Rut mut4 proved to be the best transglycosylating tool in the entire series, showing the highest trans-rutinosylation activity in the series with all acceptors tested. Its TG/H ratios ranged from 0.8 (catechol) to 3.5 (phenylethanol). The transglycosylation potential of the other three mutant variants was less prominent, ranking them between *Ao*Rut WT and *An*Rut WT, in the approximate order: mut5 > mut3 > mut6. In the reactions with *An*Rut mut3, a lower enzyme concentration was used (50% less than that with other enzymes) because mut3 precipitated at higher concentrations. Mut3 showed the highest transglycosylation activity with phenylethanol and pentanol (TG/H = 0.4–0.5), followed by *p*NP and catechol (TG/H = 0.3). *An*Rut mut5 showed the highest transglycosylation activity with catechol (TG/H = 0.7) and phenylethanol (TG/H = 0.5), while the activity with other acceptors was lower (TG/H = 0.2). The least efficient mutant variant, *An*Rut mut6, showed low trans-rutinosylation activity with all acceptors (TG/H = 0.05–0.15), similar to *An*Rut WT.

Further pilot experiments on the trans-rutinosylation activity of the enzymes *Ao*Rut WT and *An*Rut WT with rutin as a donor and several challenging acceptors, namely, cholic (**17**) and deoxycholic (**18**) acid and flavonoids—quercetin (**13**), luteolin (**14**), myricetin (**15**), and taxifolin (**16**)—yielded no detectable products as determined by HPLC and LC-MS (Supporting Information, Figures S8 and S9). Therefore, we hypothesize that these compounds do not serve as acceptors for rutinosidase-catalyzed transglycosylations under the given conditions, mainly due to their bulkiness, and, in the case of acid acceptors, also due to the negatively charged carboxyl group with a large hydration shell.

### *In Silico* Analysis of the Side Tunnel in *An*Rut Mutant Variants by Tunnel-Mapping Tools

Possible pathways of transglycosylation acceptors to the active site through the side tunnel or groove were explored qualitatively using the structures of the rutinosyl-enzyme intermediates derived from MD simulations and tunnel-mapping tools (Supporting Information, Figure S21A). The side tunnel or groove was generally wide enough to allow passage of the relatively small acceptors tested in this study. In *An*Rut WT, residues F221, F261, and Y284 are located in one segment of the side tunnel,^4^ where their side chains interact with the aglycone of the bound substrate and form the +1 subsite (Supporting Information, Figure S20B,D). These residues also formed favorable interactions with acceptors before they reached the empty aglycone binding site (the +1 subsite; Supporting Information, Figure S21B), thus playing a dual role in both substrate and acceptor binding. Binding energy profiles (Supporting Information, Figure S21A) showed drops in the free energies of binding for aromatic compounds, such as phenylethanol and *p*-nitrophenol, at a distance of 0.45–0.5 nm from the active site (close to F221, F261, and Y284 for *An*Rut WT and mut3), indicating less favorable access to the active site using the side tunnel. Additionally, modeling of the passage of these acceptors through the tunnel revealed their unfavorable orientation for the nucleophilic attack on the glycosyl-enzyme intermediate before deglycosylation (Supporting Information, Figure S21B–E). In contrast, due to the more solvent-exposed side groove, *Ao*Rut and mut5 exhibited unhindered access for transglycosylation acceptors without substantial drops in the free energy of binding and with impaired access of water molecules to the glycosyl-enzyme intermediate (Supporting Information, Figures S21A and S22A), which are all favorable conditions for transglycosylation. The Y286A mutation in mut4 created a side groove, providing additional space for bulkier transglycosylation acceptors including *p*NP (Supporting Information, Figure S21C,E) with more favorable free energies of binding close to the covalent glycosyl-enzyme intermediate (Supporting Information, Figure S21A). The glycosyl-enzyme intermediate was reoriented in mut4 compared with *An*Rut, with changes in its water network (Supporting Information, Figure S22B). In mut3, a drop in the free energies of binding for phenylethanol, catechol, and *p*NP at a distance of 0.7 nm from the active site indicated the possibility of acceptor binding too far from the glycosyl-enzyme intermediate for efficient transglycosylation (Supporting Information, Figure S21A). The energy profile for transglycosylation acceptors approaching the active site in mut6 was not energetically favorable for all acceptors (Supporting Information, Figure S21A).

## Discussion

Two of the mutant variants, mut1 and mut2, were not heterologously produced in *P. pastoris*. We hypothesize that the introduced point mutations destabilized the protein to such an extent that proper folding was impossible and that the production system of *Pichia* degraded these proteins *in situ*. This hypothesis was supported by *in silico* analysis of the impact of the introduced mutations. The main reason for mut1 instability may be the introduction of the bulky hydrophobic residue *I*222, resulting in the protein instability in the absence of a hydrophobic environment. Hydrophobic residues, such as Ile, Val, or Leu, are usually clustered in hydrophobic patches to ensure protein stability,^38^ but there are no candidates for clustering close to *I*222 in mut1. The instability of mut2 was most likely associated with its high local flexibility, accompanied by changes in nucleophile orientation. In mut7, deletion of the loop ^216^TNMVVFGTPL^225^ led to the formation of a strongly water-exposed active site with very flexible neighboring regions, which may have caused the reduced activity of mut7.

MD simulations suggested that mutations in the side tunnel may substantially influence substrate orientation and flexibility during enzyme–substrate interactions. The substrates with a bulky leaving group, such as quercetin, showed an increased number of aglycone-mediated interactions and decreased solvation. Stronger interactions increased substrate affinity, but slower release of the leaving group after the attack by the catalytic nucleophile may impair enzyme-specific activity and the transglycosylation potential. The substrates with a *p*NP leaving group behaved differently, mainly because the small *p*NP group rapidly left the +1 subsite, not obstructing the passage of the acceptor to the glycosyl-enzyme intermediate as quercetin did in mut6. *p*NP-Glc also interacted differently with the active site residues, affecting hydrolysis.

In the structure of *An*Rut, aromatic residues in the aglycone binding (+1 subsite) segment of the side tunnel, such as F221 and Y286, play a pivotal role in stabilizing substrates, particularly their aglycones (*e.g*., quercetin), through hydrophobic and π-stacking interactions.^2^ Mutations affecting these aromatic residues, such as F221A or Y286A in *An*Rut mut3 and mut4, disrupted aglycone binding and accelerated its exit during transglycosylation, favoring transglycosylation at the expense of hydrolytic efficiency. *Ao*Rut WT maintained better catalytic balance than *An*Rut WT, achieving higher TG/H ratios without compromising hydrolytic capability, making it a more versatile biocatalyst. The free energies of binding, the number of hydrogen bonds, and the stacking interactions of substrates with different aglycones clearly show that the aglycone plays an important role in substrate stabilization in the studied rutinosidases.^2,3^

MD simulations and tunnel-mapping tools revealed that the side tunnel of *An*Rut is dynamic and, in some variants, it has a high probability of being open, forming a groove as in *Ao*Rut or mut5. This behavior may change with different substrates; e.g., in mut6, the side tunnel opened upon binding substrates with bulkier aglycones, which allowed easier water access to the transglycosylation donor and reduced transglycosylation yields. In mut4, the Y286A mutation made its side tunnel groove-like, providing additional space for larger transglycosylation acceptors. The calculated energy profiles for the passage of the transglycosylation acceptor through the side tunnel or groove showed especially favorable energies for phenylethanol, pentanol, and catechol acceptors with *An*Rut mut4 or *Ao*Rut. For other acceptors, the side tunnel may not always be a clearly preferable entry route to the active site, as indicated by the energy barriers.

Differences in transglycosylation activities are often associated with the competition between water and the transglycosylation acceptor for access to the covalently bound glycosyl-enzyme intermediate.^37^ We hypothesize that mutational perturbations in the active site cavities of mut3, mut4, and mut5 led to the improved TG/H activity ratios due to a combination of advantageous factors: (*i*) increased acceptor accessibility through a less occluded active-site entrance; (*ii*) facilitated release of the leaving group (quercetin) after formation of the intermediate due to reduced stacking interactions; (*iii*) reduced solvent accessibility of the anomeric center of the intermediate; and (*iv*) a greater number of catalytically incompetent water molecules around the intermediate, slowing down hydrolysis,^41^ consistently with the previously proposed mechanisms for improving TG/H activity ratios.^42–44^ Our data indicate that mut4, which has the highest TG/H ratios for all investigated acceptors, makes the best use of all four transglycosylation-enhancing factors. Mut5 appears to operate mainly through factors (*i*) and (*iv*). However, an enlarged active-site entrance, as in mut5, may greatly reduce acceptor affinity, complicating acceptor fixation before attacking the anomeric center of the covalent glycosyl-enzyme intermediate. Mut3 may rely on a more open side entrance (*i*) along with an increased number of water molecules in the active site, which crowd the attack of the catalytic water on the anomeric center of the intermediate (*iv*). Similarly, the higher transglycosylation activity of *Ao*Rut WT compared with *An*Rut WT is likely to result from a combined effect of these factors, with the exception of (*iv*). A more flexible loop framing the tunnel in mut6 may restrict the leaving group from escaping the active site, impairing transglycosylation. A comprehensive understanding of the properties of the active-site entrance and +1 subsite provides a foundation for further rutinosidase engineering to enhance transglycosylation activity.

In conclusion, the *An*Rut WT side tunnel with its +1 subsite segment favors hydrolysis over transglycosylation. Reshaping of the side tunnel into a groove to facilitate acceptor access and aglycone release as well as modulating water access to the glycosyl-enzyme intermediate may be the reasons for increased transglycosylation in *An*Rut mut3, mut4, and mut5. In this work, we observed a strong correlation between the transglycosylation activity and +1 subsite interactions, as well as active-site entry geometries. We hypothesize that replacing aromatic aglycone binding residues and moderately widening the active-site entry with small hydrophobic residues are straightforward approaches to improve transglycosylation activities in GH5_23 rutinosidases for rutinosylation or glucosylation of target acceptors.

## Supplementary Material



## Notes

Detailed experimental procedures and characterization data: synthesis of *p*-nitrophenyl rutinoside (**5**), and its NMR, HRMS, and HPLC characterization; preparation and characterization of *An*Rut mutant variants (primer sequences, purification protocols, SDS–PAGE analyses, and kinetic curves); HPLC analyses of transglycosylation reactions; and additional molecular modeling data and figures (PDF)
