# Supplementary material for: Fungal Rutinosidase—Engineering of a Side Tunnel Steers Its Transglycosylation Potential
Source: J Agric Food Chem. 2026 May 18;74(21):16646–56. doi: 10.1021/acs.jafc.6c02816 (PMC13237774; doi:10.1021/acs.jafc.6c02816)
Supplement: Supplementary file 1 [file jf6c02816_si_001.pdf]

Supporting Information

Fungal Rutinosidase – Engineering of Side Tunnel Steers Its Transglycosylation Potential

Lucie Petrásková[a], Michael Kotik[a], Natalia Kulik [a], Andrea Vopálenská [a,b], Anna Šidáková[a,c], Katerina Brodsky[a], Vladimír Křen[a], Pavla Bojarová[a,d,*]

[a] Institute of Microbiology of the Czech Academy of Sciences, Vídeňská 1083, CZ 142 00, Prague 4, Czech Republic

[b] Department of Organic Chemistry, Faculty of Science, Charles University, Albertov 6, CZ 128 00 Prague 2, Czech Republic.

[c] Department of Analytical Chemistry, Faculty of Science, Charles University, Albertov 6, CZ 128 00 Prague 2, Czech Republic.

[d] Department of Health Care Disciplines and Population Protection, Faculty of Biomedical Engineering, Czech Technical University in Prague, nám. Sítná 3105, CZ 272 01 Kladno, Czech Republic

* Phone: (+420)-296-442-360; Email: bojarova@biomed.cas.cz

Content

1. Synthesis and Characterization of *p*NP-Rutinoside

2. Preparation and Characterization of *An*Rut Mutant Variants

3. Transglycosylation Potential of Mutant and Wild-type Enzymes

4. Molecular Modeling

5. References

6. Abbreviations Used

1. Synthesis and Characterization of *p*NP-Rutinoside

6-*O*-(α-L-rhamnopyranosyl)-D-glucopyranose heptaacetate (2)

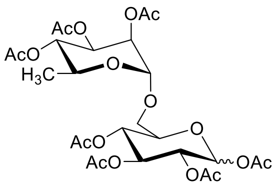

Acetic anhydride (12 mL, 124.2 mmol, 9.0 equiv) was added to a stirred solution of rutinose **1** (4.5 g, 13.8 mmol, 1.0 equiv) in anhydrous pyridine (20 mL) at 0 °C (ice/water bath). The reaction mixture was stirred at room temperature overnight (12 h). After full consumption of starting rutinose **1** (monitored by TLC, cyclohexane/EtOAc = 1/2), the reaction mixture was cooled to 0 °C (ice/water bath) and carefully (exothermic reaction) quenched by slow addition of methanol (20 mL). The resulting solution was concentrated, the residue was dissolved in EtOAc (40 mL), washed with 1M HCl (2 × 40 mL), water (1 × 40 mL), aqueous solution of $NaHCO_3$ (1 × 40 mL), and brine (1 × 40 mL). The organic phase was dried over Na_2SO_4. After filtration of drying agent, solvents were removed under reduced pressure, furnishing acetate **2** as a colorless syrup (6.8 g, 80%). The NMR spectra were identical with the published ones and were in accord with the compound structure.[1]

2,3,4-Tri-*O*-acetyl-4-nitrophenyl 6-*O*-(2′,3′,4′-tri-*O*-acetyl-α-L-rhamnopyranosyl)-β-D-glucopyranoside (4)

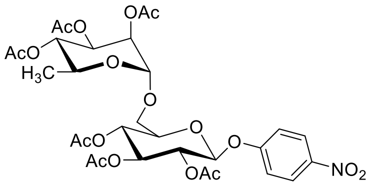

Hydrogen bromide solution in acetic acid (33 wt%, 18.0 mL, 99.9 mmol, 10.0 equiv) was added dropwise to a stirred solution of acetate **2** (6.2 g, 9.99 mmol, 1.0 equiv) in anhydrous CH_2Cl_2 (40 mL) and acetic anhydride (1.5 mL, 19.9 mmol, 2.0 equiv) at 0 °C (water/ice bath). At this temperature, the reaction mixture was stirred for 30 minutes and then was heated up to room temperature and left to stir for 2 h. After full consumption of starting peracetate **2** (monitored by TLC, cyclohexane/EtOAc = 1/2), the reaction mixture was cooled to 0 °C (water/ice bath) and was diluted with CH_2Cl_2 (40 mL) and water (90 mL). The organic phase was washed with water (5 × 90 mL), aqueous solution of $NaHCO_3$ (1 × 90 mL), and brine (1 × 90 mL). The organic phase was dried over Na_2SO_4. After filtration of drying agent, solvents were removed under reduced pressure, furnishing bromide **3** as a colorless syrup (6.4 g, 99%), which was used in the next reaction step without further purification.

The solution of bromide **3** (6.4 g, 9.98 mmol, 1.0 equiv) in anhydrous pyridine (40 mL) was dropwise added to the stirred solution of 4-nitrophenol (2.3 g, 16.9 mmol, 1.7 equiv), 3 Å molecular sieves (0.5 g) and Ag_2CO_3 (8.2 g, 29.9 mmol, 3.0 equiv) in dry pyridine (60 mL) at 0°C under argon atmosphere. The reaction mixture was stirred for 2 h at room temperature. After full consumption of starting bromide **3** (monitored by TLC, toluene/EtOAc = 2/1), the reaction mixture was filtered through Celite®, washed with EtOAc (3 × 10 mL). Filtrate was concentrated under reduced pressure. The resulting crude product was purified by column chromatography (eluting with toluene/EtOAc = 2/1), which afforded glycoside **4** (2.9 g, 42%) as a colorless syrup. The NMR spectra were identical with the published ones and were in accord with the compound structure.[2]

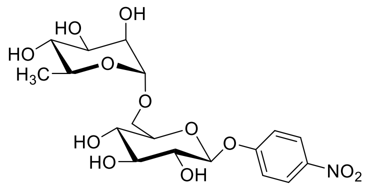

4-Nitrophenyl 6-*O*-(α-L-rhamnopyranosyl)-β-D-glucopyranoside (5)

The solution of MeONa in anhydrous MeOH (0.2M) was added dropwise to a stirred solution of glycoside **4** (2.8 g, 4.11 mmol, 1.0 equiv) in anhydrous MeOH (80 mL) at room temperature. The reaction mixture was stirred for 3 h at room temperature. After full consumption of starting glycoside **4** (monitored by TLC, toluene/EtOAc = 2/1), the reaction mixture was neutralized with Dowex® 50WX2 ion-exchange resin, filtered, and freeze-dried. The final product **5** (1.3 g, 72%) was obtained as a white solid. The NMR spectra were identical with the published ones and were in accord with the compound structure.[3]

Table S1. ^{1}**H** and ^{13}**C NMR** data (600.23 MHz for ^{1}H, 150.93 MHz for ^{13}C, CD_3OD, 30 °C) for compound **5**.

	Atom	δ_C	m.	δ_H	n_H	m.	*J* [Hz]	diagnostic HMBC
**aglycon**	***ipso-***	161.79	s	-	0	-		1^G, *meta-*
	ortho-	116.70	d	7.261	2	m	Σ*J* = 9.4	
	meta-	126.30	d	8.290	2	m	Σ*J* = 9.4	
	para-	142.84	s	-	0	-		*ortho-*
**Glc**	**1**	99.43	d	5.303	1	m		
	**2**	72.97	d	3.673	1	m		
	**3**	75.65	d	3.673	1	m		
	**4**	69.95	d	3.577	1	m		
	**5**	75.28	d	3.840	1	ddd	9.8, 6.1, 2.1	
	**6**	66.30	t	4.061	1	dd	11.5, 2.1	1^F
				3.758	1	dd	11.5, 6.1	
**Rha**	**1**	100.41	d	4.794	1	d	1.8	6^G
	**2**	70.25	d	3.938	1	dd	3.5, 1.8	
	**3**	70.47	d	3.797	1	dd	9.7, 3.5	
	**4**	72.22	d	3.432	1	dd	9.7, 9.6	
	**5**	68.86	d	3.729	1	dq	9.6, 6.3	
	**6**	16.68	q	1.192	3	d	6.3	

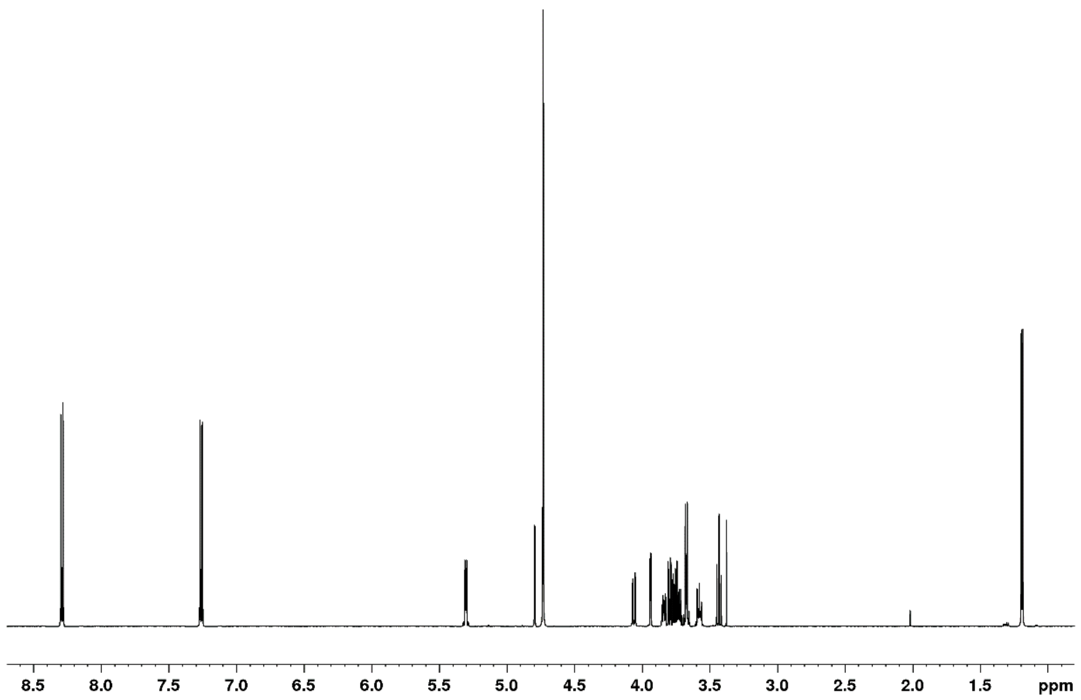

Figure S1. ^{1}H NMR spectrum of compound **5** (600.23 MHz for ^{1}H, CD_3OD, 30 °C).

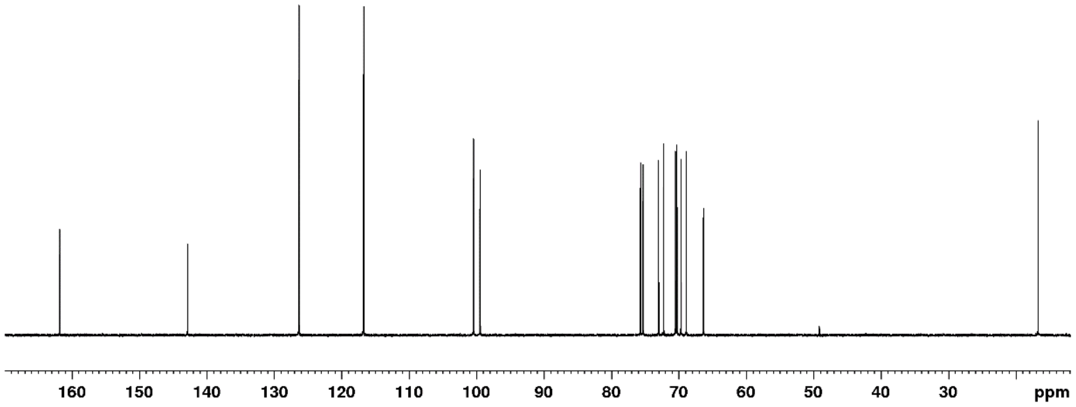

Figure S2. ^{13}C NMR spectrum of compound **5** (150.93 MHz for ^{13}C, CD_3OD, 30 °C).

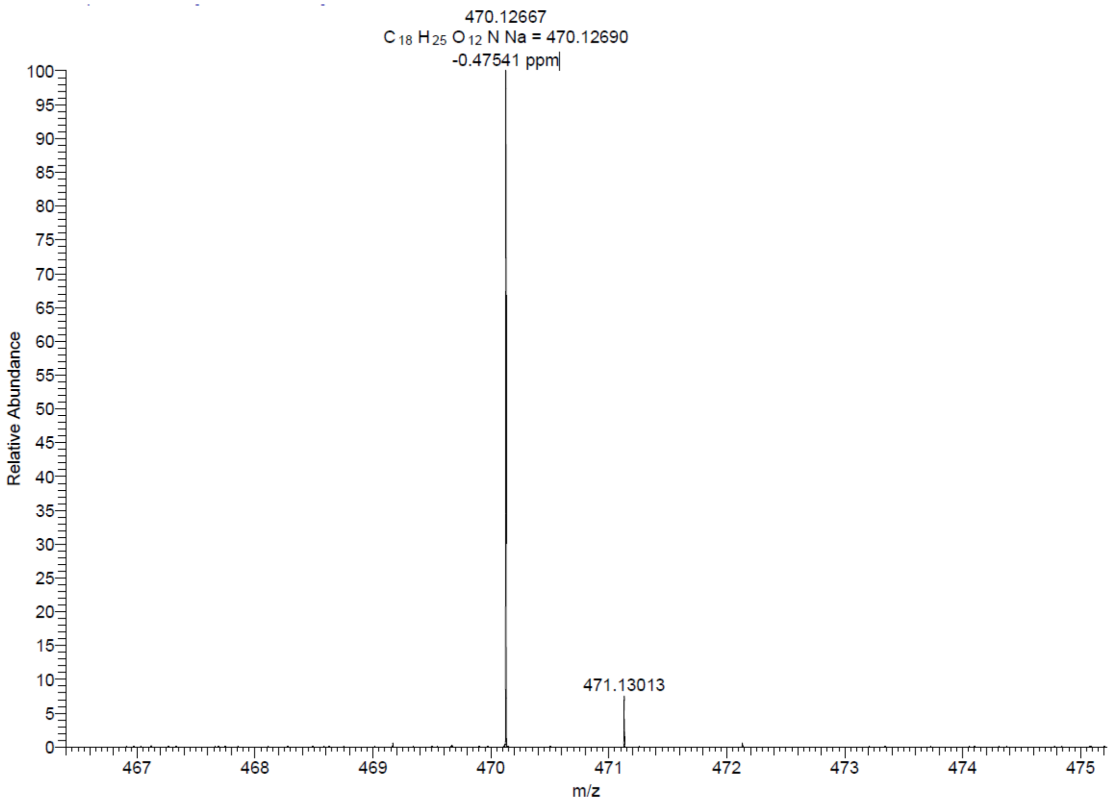

Figure S3. HRMS (ESI^+) spectrum of compound **5**: *m/z* calcd. for $C_{18}H_{25}O_{12}NNa$ $[M+Na]^+$: 470.12690, found: 470.12667.

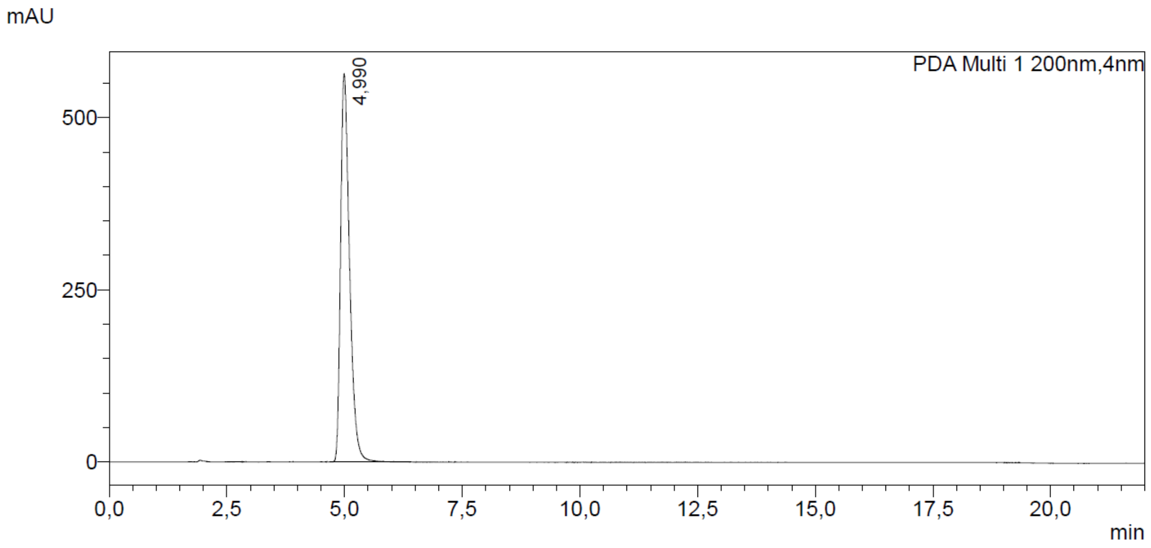

Figure S4. HPLC chromatogram of compound **5** (4.990 min, 100% purity).

2. Preparation and Characterization of *An*Rut Mutant Variants

Table S2. Sequences of primers used for the construction of *An*Rut mutants mut1, mut3, mut4, and mut6.

Mutant	Primer	Sequence
**1**	G222I-1	5'-gggcgaggggcgtgatgaagacgaccatgt
	G222I-2	5'-acatggtcgtcttcatcacgcccctcgccc
**3**	F221A-1	5'-cgaggggcgtgccggcgacgaccatgttgg
	F221A-2	5'-ccaacatggtcgtcgccggcacgcccctcg
**4**	Y286A-1	5'-gacgaggaatccatatgctcggcgtaataatgatgcgtatcgaag
	Y286A-2	5'-cttcgatacgcatcattattacgccgagcatatggattcctcgtc
**6**	M218G/V219G-1	5'-gggcgtgccgaagacgcccccgttggtgttgttgtcg
	M218G/V219G-2	5'-cgacaacaacaccaacgggggcgtcttcggcacgccc
	H288G_1	5'-ctccgacgaggaatccatacctcgtagtaataatgatgc
	H288G_2	5'-gcatcattattactacgagggtatggattcctcgtcggag

Table S3. Typical purification data of the heterologously produced wild-type rutinosidases *An*Rut and *Ao*Rut and the mutant rutinosidases *An*Rut mut3 to mut6. *p*NP-rutinoside was used as the substrate in the standard activity assay.

Enzyme	Purification step [a]	Total protein [mg]	Total activity [U]	Specific activity [U mg^{-1}]	Purification yield [%]
*Ao*Rut	Culture medium	641	1388	2.2	
	Cation exchange	101	440	4.4	32
*An*Rut	Culture medium	109	50	0.46	
	Cation exchange	11	18	1.64	36
*An*Rut mut3	Culture medium	52	n.d.	n.d.	
	Ultrafiltration	50	0.9	< 0.1	n.a.
*An*Rut mut4	Culture medium	40	n.d.	n.d.	
	Ultrafiltration	24	2.5	0.1	n.a.
*An*Rut mut5	Culture medium	52	11	0.2	
	Ultrafiltration	9.9	7.0	0.7	64
*An*Rut mut6	Culture medium	167	23	0.1	
	Cation exchange	16	7.0	0.4	30

[a] The purification was always performed from 400 mL of medium. n.d., not detectable. n.a., not applicable.

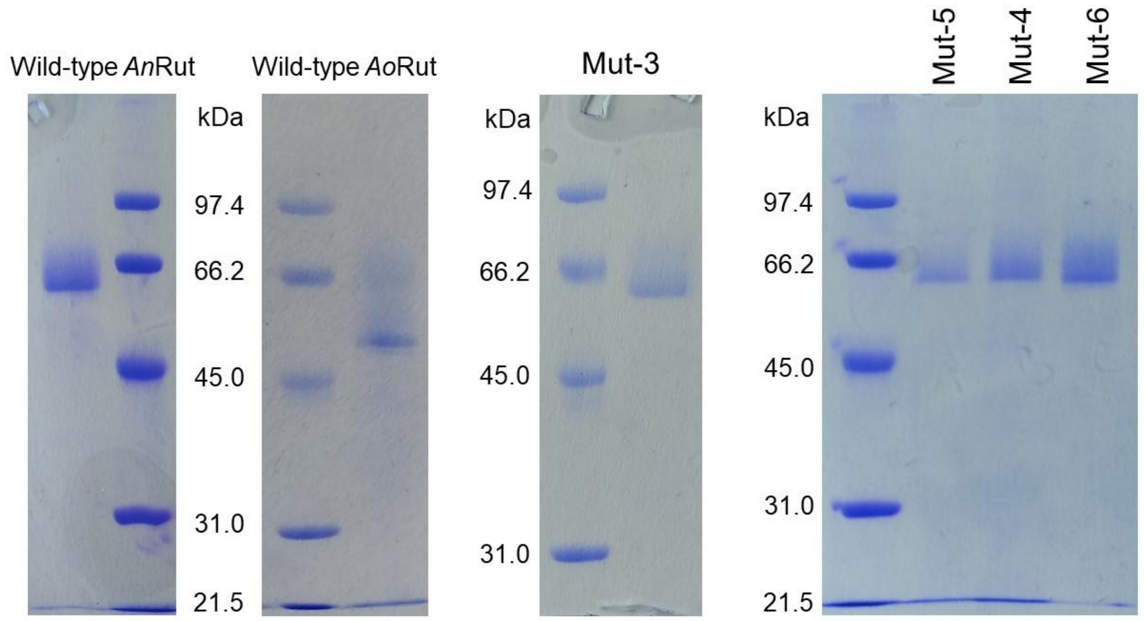

Figure S5. SDS-PAGE analysis of the heterologously produced wild-type rutinosidases *An*Rut and *Ao*Rut and the mutant enzymes mut3 to mut6. The molecular weights of the marker proteins are indicated. The slight heterogeneity of the purified samples is presumably given by slight differences in protein glycosylation, as observed previously.[4]

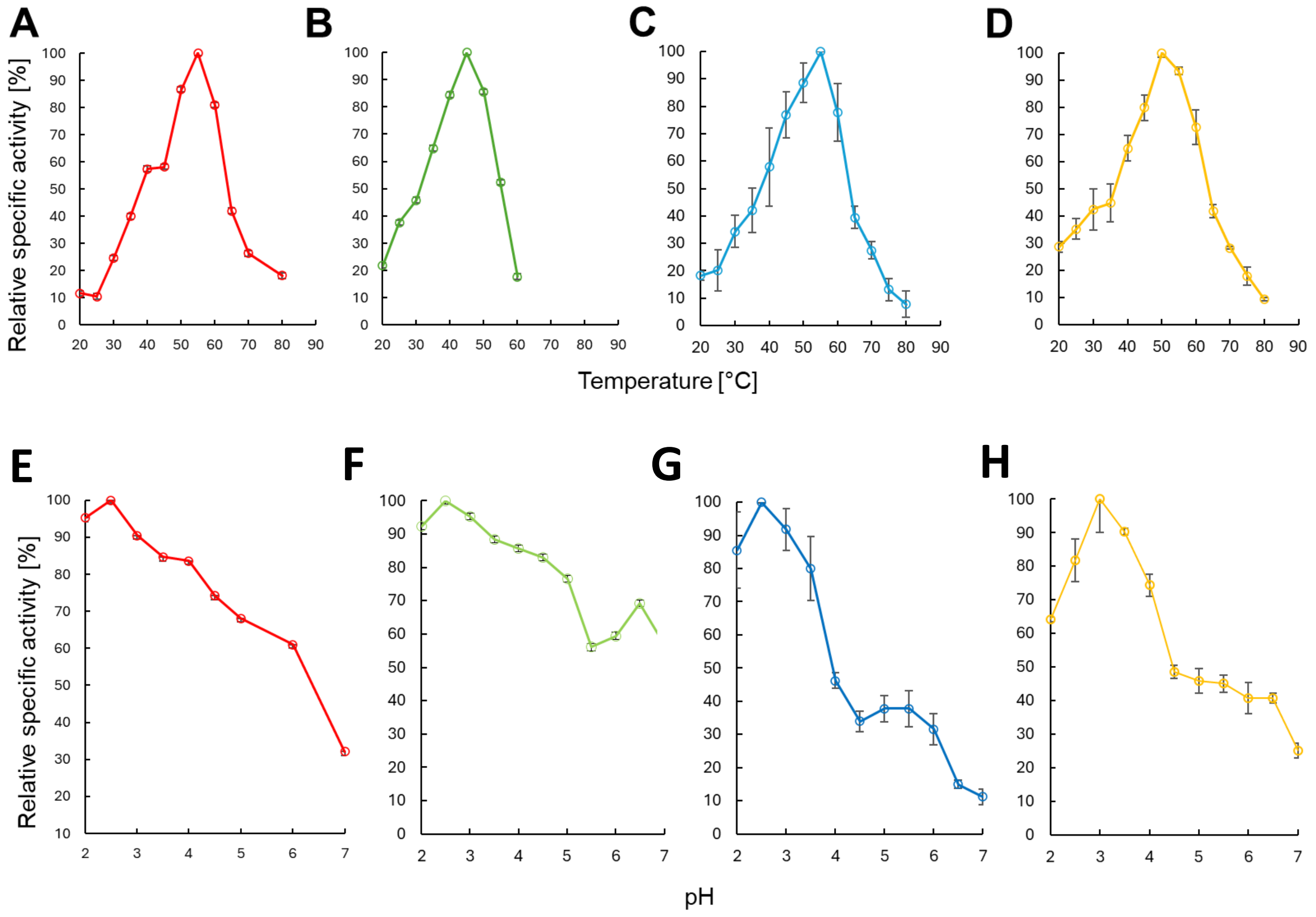

Figure S6. (**A**-**D**) pH Optima of *An*Rut mutant variants *An*Rut mut3 (**A**); *An*Rut mut4 (**B**); *An*Rut mut5 (**C**); *An*Rut mut6 (**D**); and (**E**-**F**) temperature optima of *An*Rut mutant variants *An*Rut mut3 (**E**); *An*Rut mut4 (**F**); *An*Rut mut5 (**G**); *An*Rut mut6 (**H**).

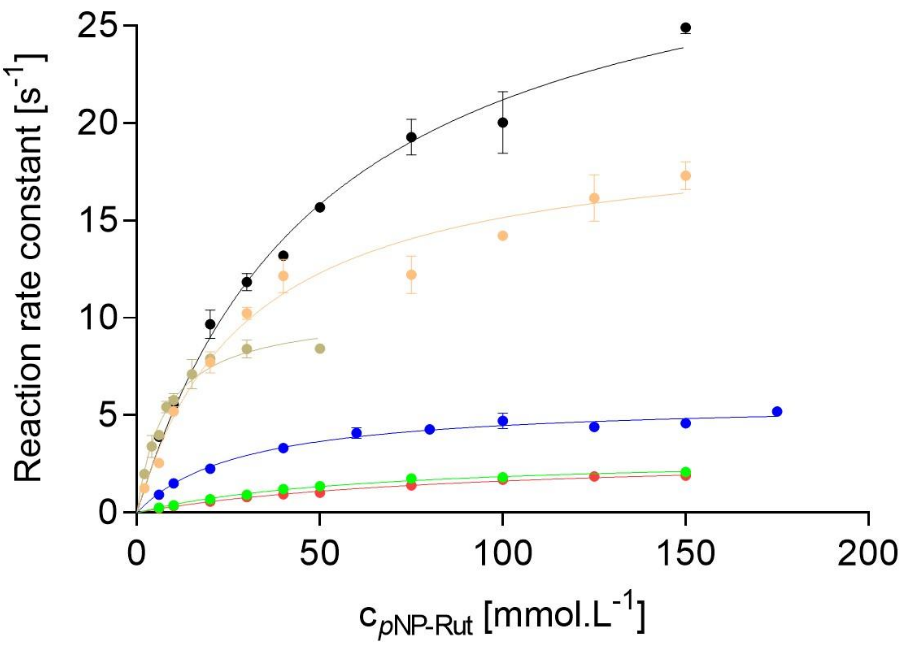

Figure S7. Kinetic parameters of rutinosidase variants. *An*Rut WT in black; *Ao*Rut WT in grey; *An*Rut mut3 in red, *An*Rut mut4 in green, *An*Rut mut5 in blue, *An*Rut mut6 in salmon.

3. Transglycosylation Potential of Mutant and Wild-type Enzymes

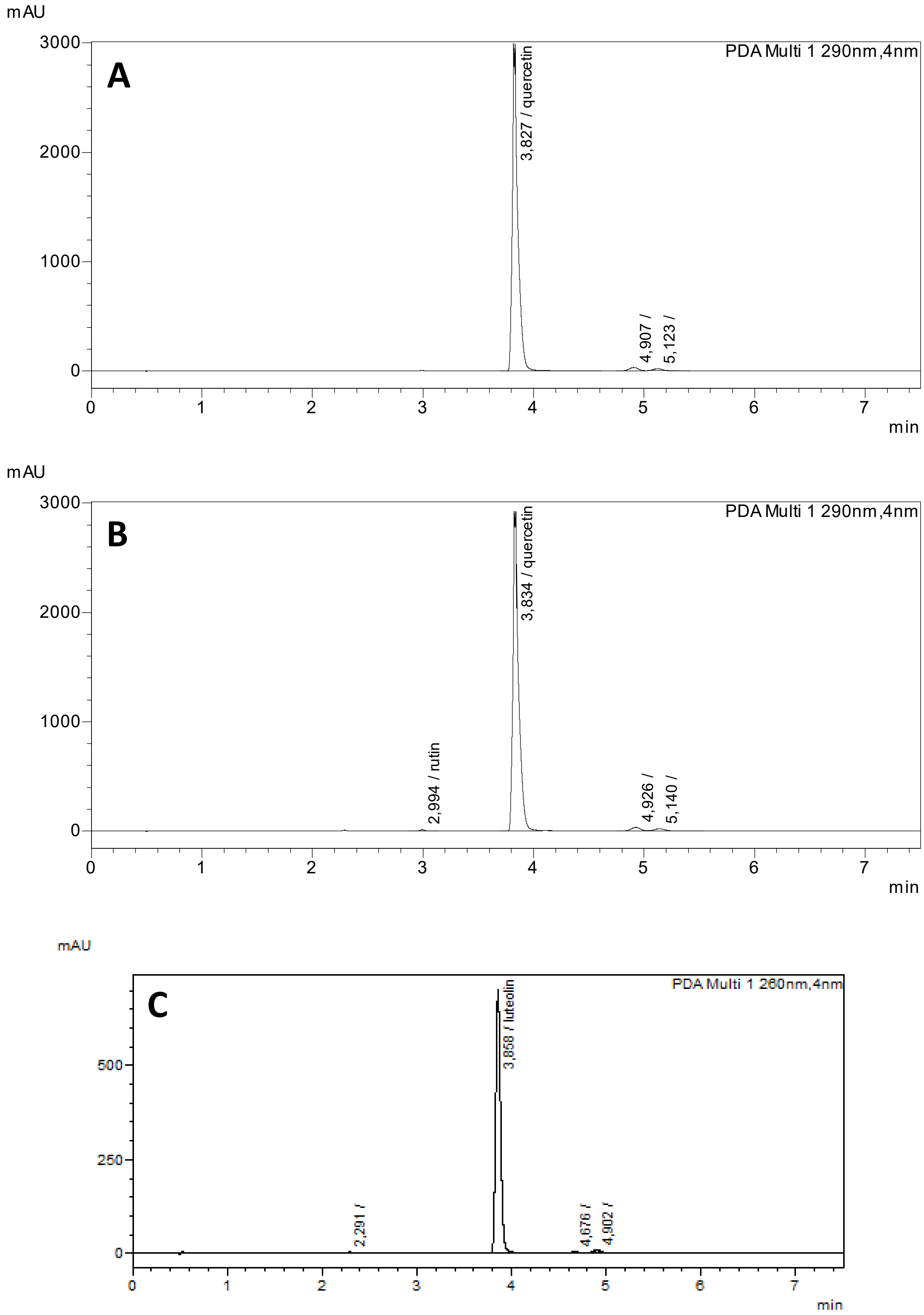

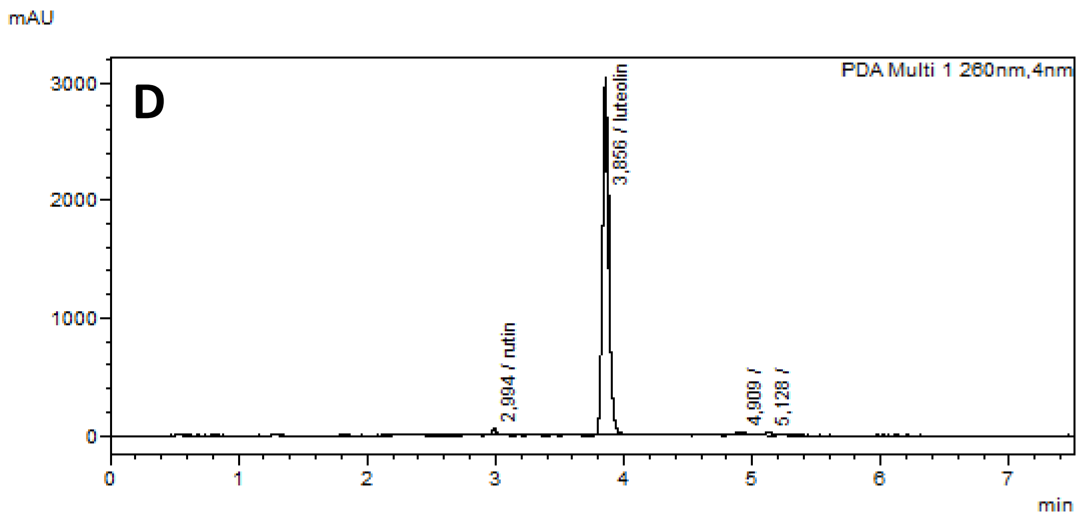
mAU
D
PDA Multi 1 260nm,4nm
3000
2000
1000
0
2,994 / rutin
3,856 / luteolin
4,909 /
5,128 /
0
1
2
3
4
5
6
7
min

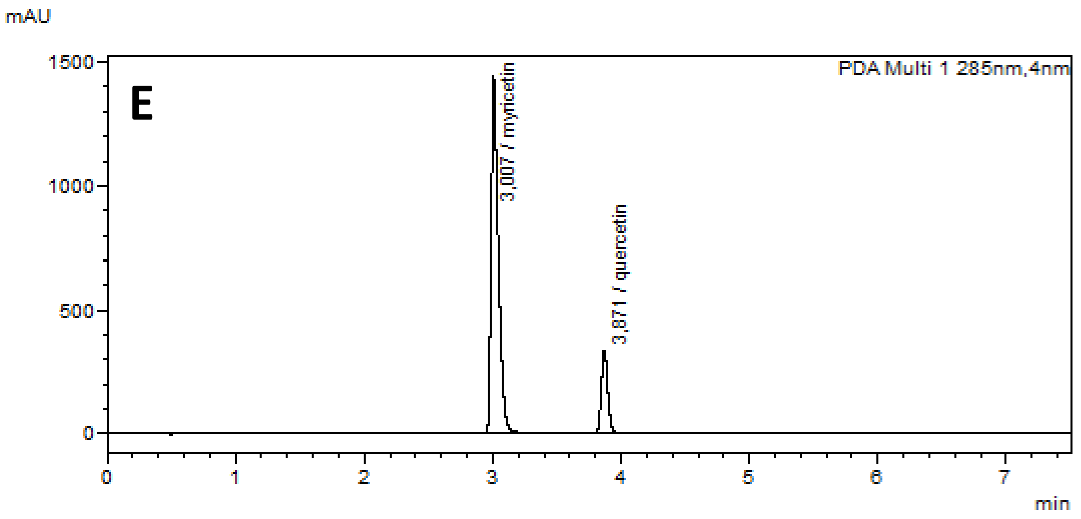
mAU
E
PDA Multi 1 285nm,4nm
1500
1000
500
0
3,007 / myricetin
3,871 / quercetin
0
1
2
3
4
5
6
7
min

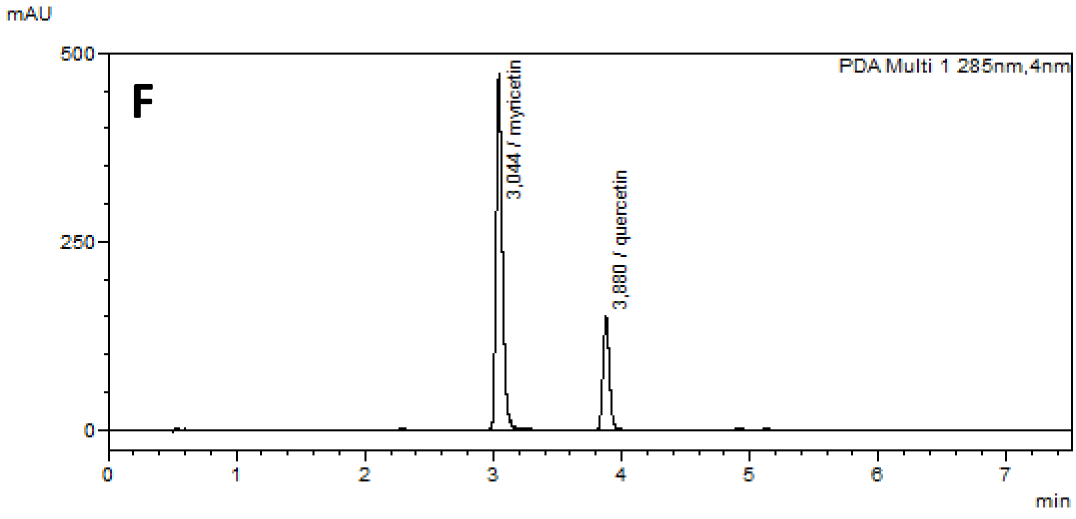
mAU
F
PDA Multi 1 285nm,4nm
500
250
0
3,044 / myricetin
3,880 / quercetin
0
1
2
3
4
5
6
7
min

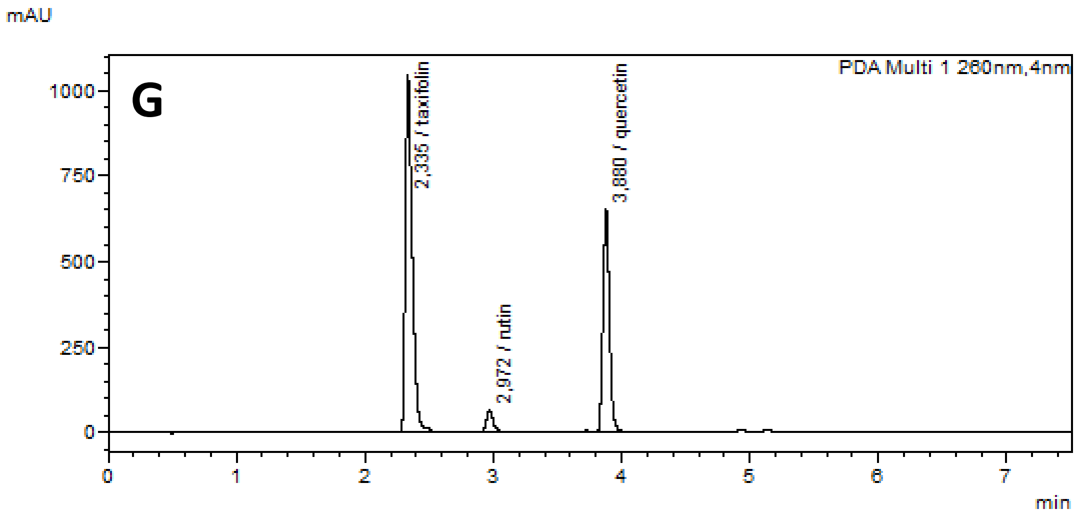

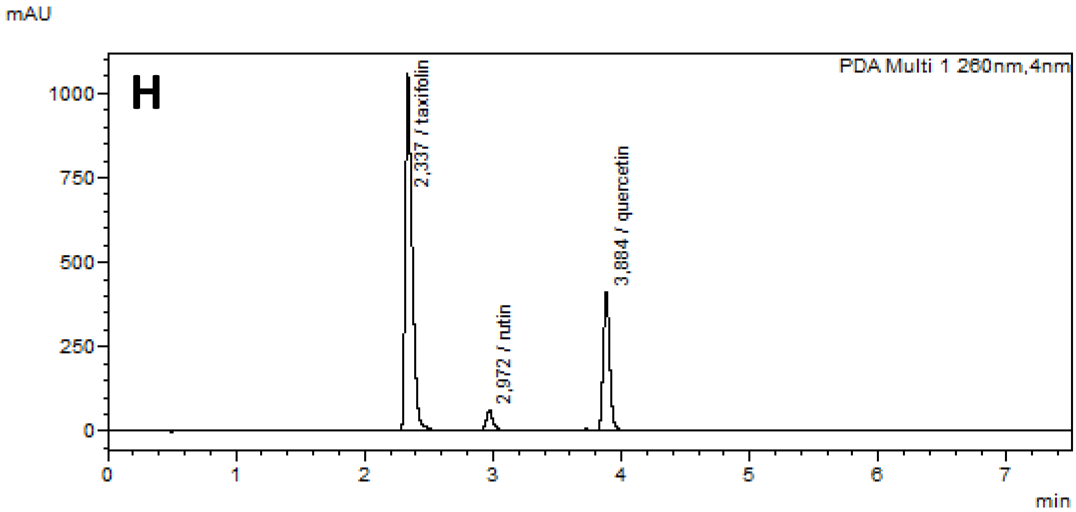

Figure S8. HPLC chromatogram of transglycosylation reaction (after 72h) with *An*Rut and *Ao*Rut with rutin as a donor and quercetin (**13**; A, B), luteolin (**14**; C, D), myricetin (**15**; E, F), and taxifolin (**16**; G, H), as acceptors. The chromatographic conditions are specified in the main text. Luteolin and quercetin have the same retention time and are both present in the luteolin peak in chromatograms C and D.

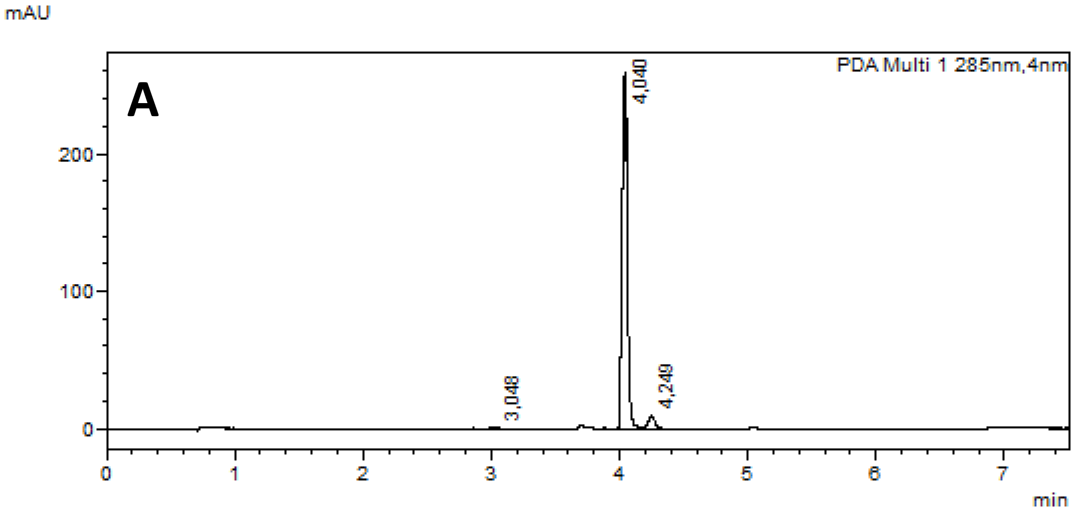

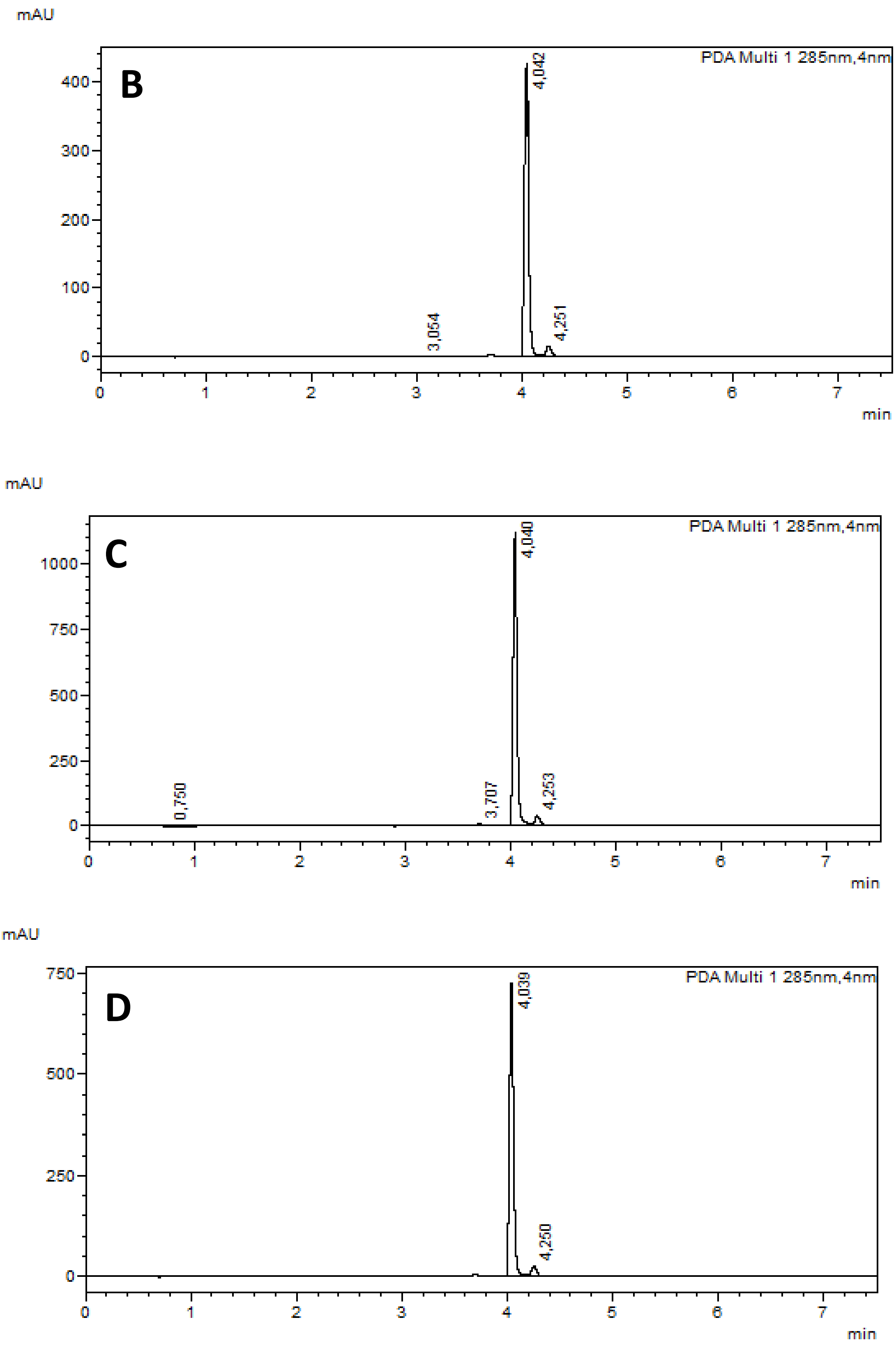

Figure S9. HPLC chromatogram of pilot transglycosylation reactions (after 24h) with *An*Rut and *Ao*Rut with rutin as a donor and cholic acid (**17**; panels A, B), and deoxycholic acid (**18**; panels C, D) as acceptors. The chromatographic conditions are specified in the main text. The expected product was not observed.

Table S4 The transglycosylation potential of the mutant and WT enzymes in a series of analytical reactions, in which the donor was rutin and the acceptor was pentanol (**9**), phenylethanol (**10**), catechol (**11**), or *p*-nitrophenol (**12**), respectively.

	**TG/H***															
**Acceptor**	pentanol (**9**)				phenylethanol (**10**)				catechol (**11**)				*p*-nitrophenol (**12**)			
**Time [h]**	2	3	24	120	2	3	24	120	2	3	4	120	2	3	24	120
**Variant**																
*Ao*Rut WT	0.60	0.51	0.46	n.d.	0.88	0.90	0.90	0.87	0.52	0.51	0.47	0.45	0.13	0.12	0.15	0.13
*An*Rut WT	0.12	0.11	0.08	0.15	0.09	0.08	0.05	0.05	0.03	0.03	0.02	0.01	0.10	0.15	0.10	0.10
mut3	0.35	0.36	0.07	0.04	0.48	0.35	0.17	0.03	0.28	0.20	0.10	0.10	0.23	0.30	0.24	0.21
mut4	1.11	1.37	0.96	0.75	2.57	2.54	3.33	3.00	0.76	0.57	0.47	0.80	1.43	1.53	1.26	1.10
mut5	0.26	0.22	0.22	n.d.	0.46	0.49	0.44	0.48	0.76	0.47	0.41	0.43	0.17	0.16	0.16	0.16
mut6	0.19	0.16	0.17	n.d.	0.13	0.12	0.08	0.08	0.13	0.08	0.03	0.02	0.06	0.04	0.06	0.05

* As a representative parameter of transglycosylation potency, we calculated the molar ratio of the respective transglycosylation product and the hydrolytic product rutinose in the reaction mixture at the same reaction time point (2, 3, 24, and 120 h). This transglycosylation/hydrolysis ratio (TG/H) can be interpreted as the ratio of transglycosylation (TG) to hydrolytic (H) activities in the reaction. A TG/H ratio equal to 1 corresponds to the extent of transglycosylation equal to hydrolysis in the reaction. n.d. means not determined.

4. Molecular Modeling

Table S5. Frequency of interactions during the last 20 ns of molecular dynamics simulation (MD) (80-100 ns), calculated by GetContacs for *An*Rut WT and *Ao*Rut WT with docked rutin. Maximum frequency is 1 (always present), minimum - 0 (never formed). The amino acid numbering used here and throughout the article for *Ao*Rut WT is based on the protein structure pdb ID: 6la0. To obtain the number corresponding to the complete sequence in the UniProt database (UniProtKB accession No.: A0A1S9DRB1), add 19.

A. Hydrogen bonds

***An*Rut residue**	***An*Rut, frequency**	***Ao*Rut, residue**	***Ao*Rut, frequency**
	**Interaction with aglycone (quercetin)**		
Glu50	*0.90*	Glu31	*0.31*
Thr163	*0.42*	Thr144	*0*
Ile164	*0.25*	Ile145	*0*
Glu210	*0.65*	Glu191	*0.96*
Met218	*0*	Met198	*0.03*
Phe221	*0*	Phe201	*0.04*
His282	*0*	His262	*0.64*
Tyr284	*0.02*	Tyr264	*0*
Tyr286	*0.02*	Phe266	*0*
Glu287	*0.84*	Glu267	*0.16*
HIS:288	*0.03*	Arg268	*0.003*
**TOTAL**	***3.13***		***2.14***
	**Interaction with -1 carbohydrate**		
Glu50	*0*	Glu31	*0.71*
His153	*0.64*	His134	*0.64*
Glu210	*0.73*	Glu191	*0.23*
His282	*0.01*	His262	*0.21*
Tyr284	*0.74*	Tr264	*0.007*
Glu319	*0.10*	Glu298	*0.10*
Trp354	*0.06*	Trp333	*0.21*
**TOTAL**	***3.18***		***3.03***
	**Interaction with -2 carbohydrate**		
Gln370	*0.77*	Gln349	*0.85*
Thr52	*0.27*	Thr33	*0.74*
Tyr286	*0.05*	Phe266	*0*
Glu50	*0.03*	Glu31	*0*
Ser51	*0.003*	Ser32	*0*
Gln323	*0.07*	Gln302	*0*
Trp374	*0.01*	Trp353	*0*
**TOTAL**	***1.12***		***1.16***

B. Hydrophobic interactions

*An*Rut residue	*An*Rut, frequency	*Ao*Rut, residue	*Ao*Rut, frequency
	**Interaction with aglycone (quercetin)**		
Leu162	*0.35*	Leu143	*0.41*
Ile164	*0.19*	Ile145	*0.17*
Phe221	*0.20*	Phe201	*0.57*
Phe261	*0.01*	Phe41	*0.39*
Tyr286	*0*	Phe266	*0.26*
**TOTAL**	***0.75***		**1.80**
	**Interaction with -2 carbohydrate**		
Ile53	0.09	Ile34	0
Phe358	0.7	Phe266	0.94
Trp374	0.43	Trp353	0.19
**TOTAL**	**1.22**		**1.12**

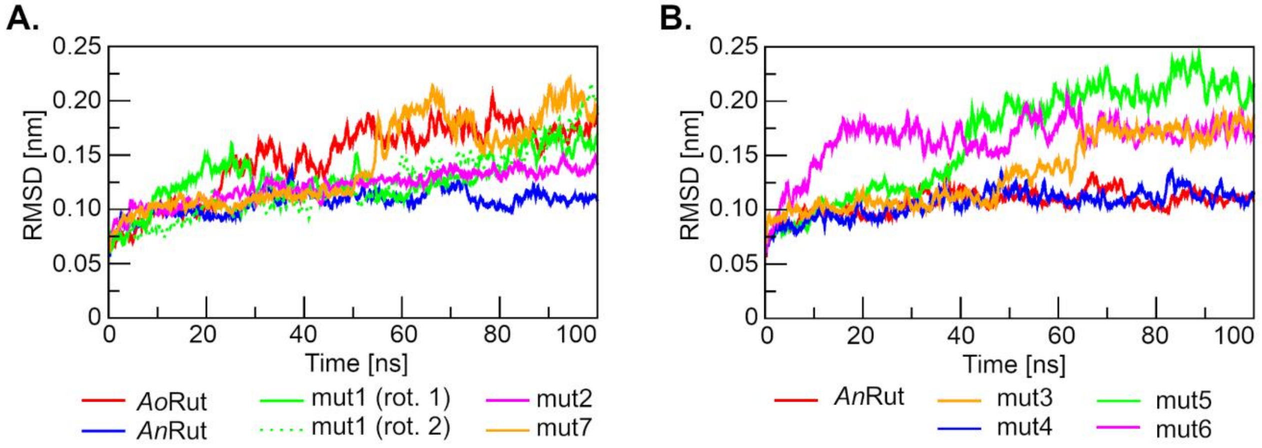

Figure S10. Root mean square deviation (RMSD) of C-α atoms of substrate-free enzymes during 100 ns of MD simulation. Since the I222 side-chain orientation was ambiguous, two I222 rotamers were modeled (rot. 1: $\chi1 = -41°$; rot. 2: $\chi1 = 75°$).

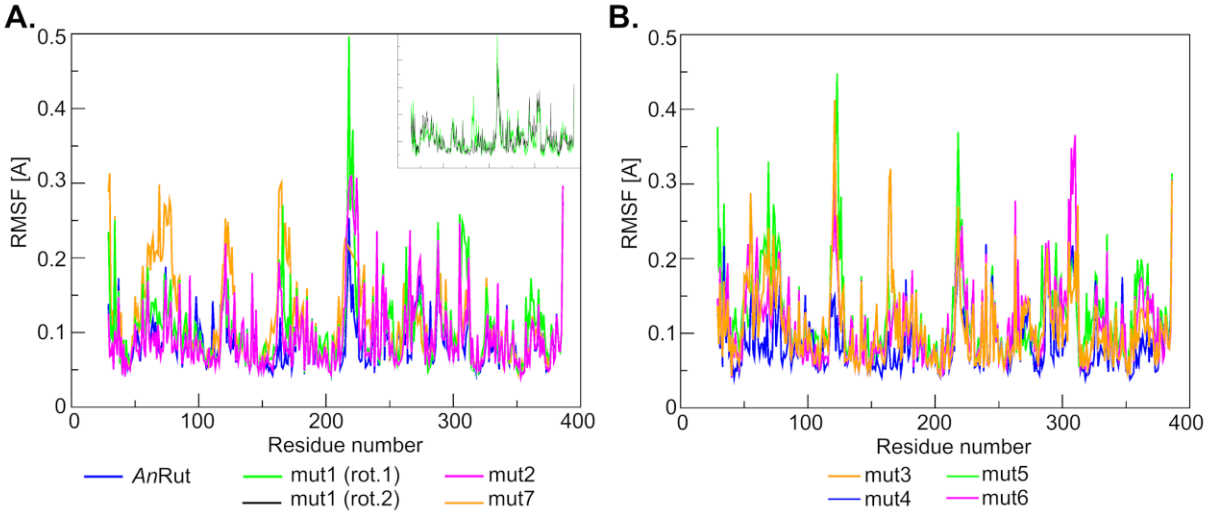

Figure S11. Root mean square fluctuation (RMSF) of amino acid residues of substrate-free enzymes during MD simulation. Mutated regions are aa 216-225, aa 261, aa 284-289, and aa 307. Inset: data for MD of *An*Rut mut1 showing various rotamers of I222. Since the I222 side-chain orientation was ambiguous, two I222 rotamers were modeled (rot. 1: $\chi1 = -41°$; rot. 2: $\chi1 = 75°$).

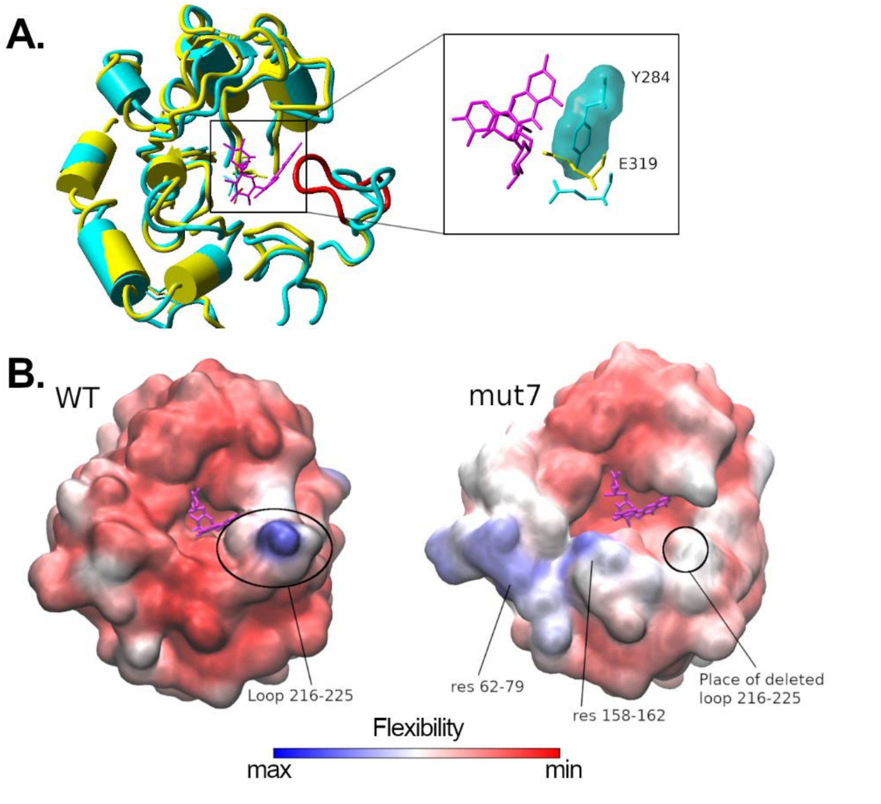

Figure S12. Changes imposed by mutations in unstable/catalytically inactive mutants *An*Rut mut2 and mut7. **A.** Changed orientation of nucleophile residue E319 in mut2. Structural alignment of substrate-free *An*Rut WT (cyan) and mut2 (yellow, with loop 216-225 in red) after 100 ns of MD simulation. The position of rutin interpolated from the docked orientation in *An*Rut WT is shown in magenta. Hydrogens are hidden. The inset shows the change in the position of catalytic nucleophile E319. The position of mutated Y284 in substrate-free *An*Rut WT (100 ns) is shown in a molecular surface representation. **B.** Changed flexibility of mut7 (left) compared with *An*Rut WT (right) after 100 ns MD. Surface is colored by β-factor expressing protein flexibility (red – low, blue – high).

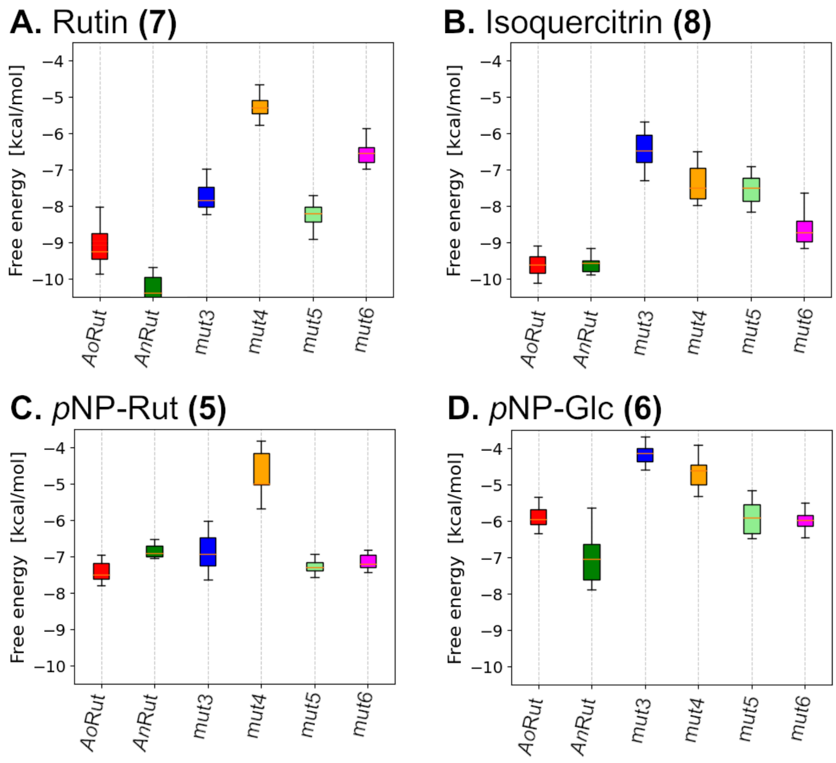

Figure S13. Free energy of binding analyzed by AutodockVina calculated for various donors (**A**, rutin; **B**, isoquercitrin; **C**, *p*NP-Rut; **D**, *p*NP-Glc) shown in a boxplot representation with data range. The red line in the middle of the boxes shows the value of the median of the dataset.

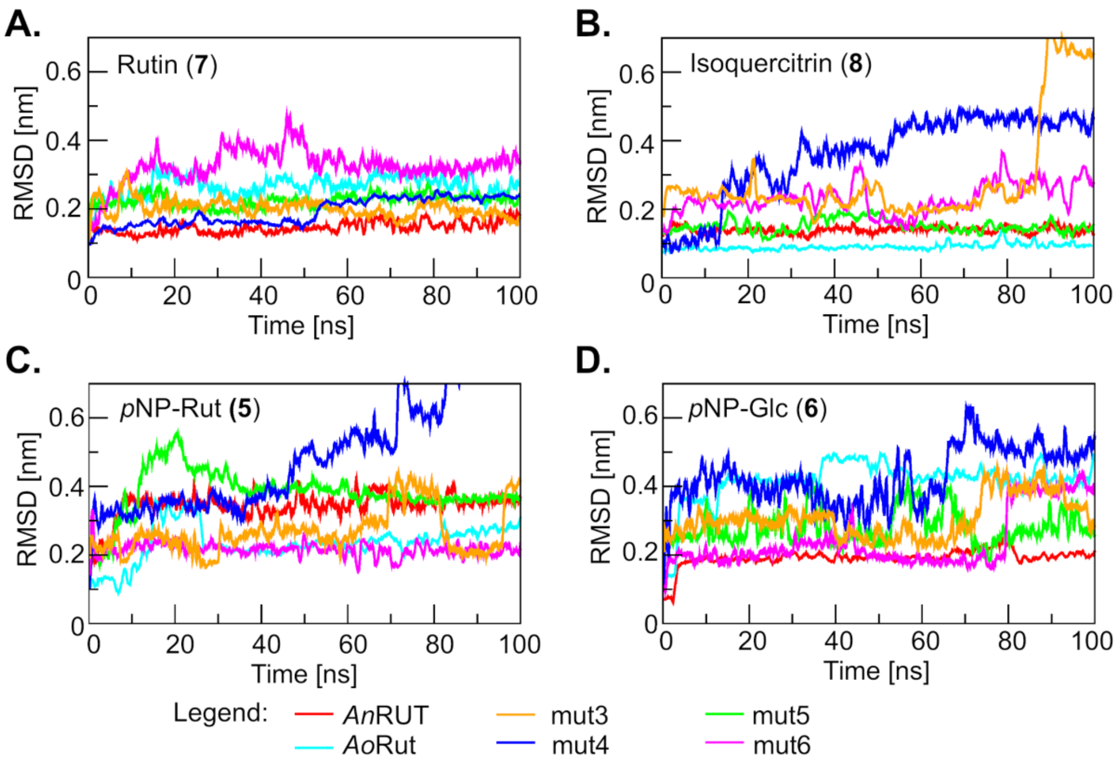

Figure S14. Root mean square deviation (RMSD) of the substrate position (**A**, rutin; **B**, isoquercitrin; **C**, *p*NP-Rut; **D**, *p*NP-Glc) with respect to the initial position. For alignment, we used the C-α atoms of the enzyme.

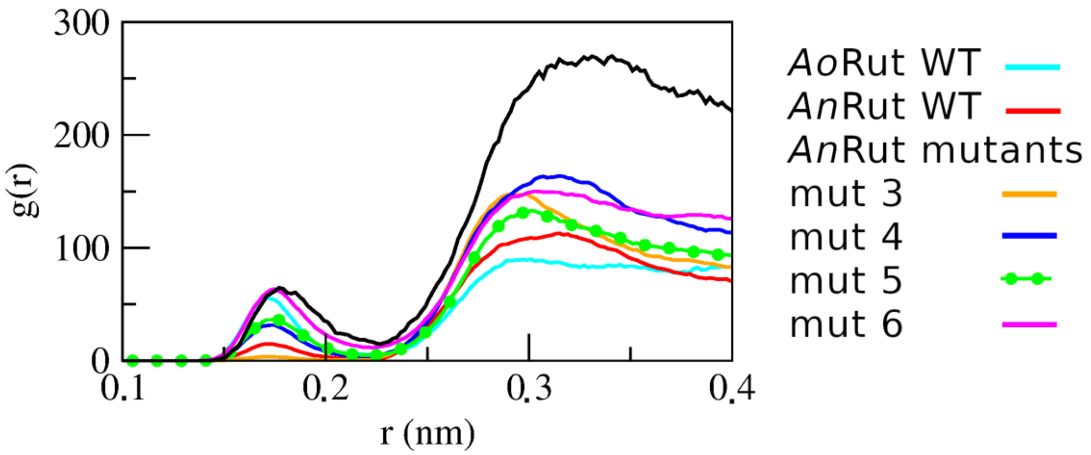

Figure S15. Radial distribution (RDF) of water molecules (oxygen atoms considered) with respect to the surface of quercetin in bound rutin substrate during MD simulation. The black curve corresponds to the quercetin solvation in bulk water.

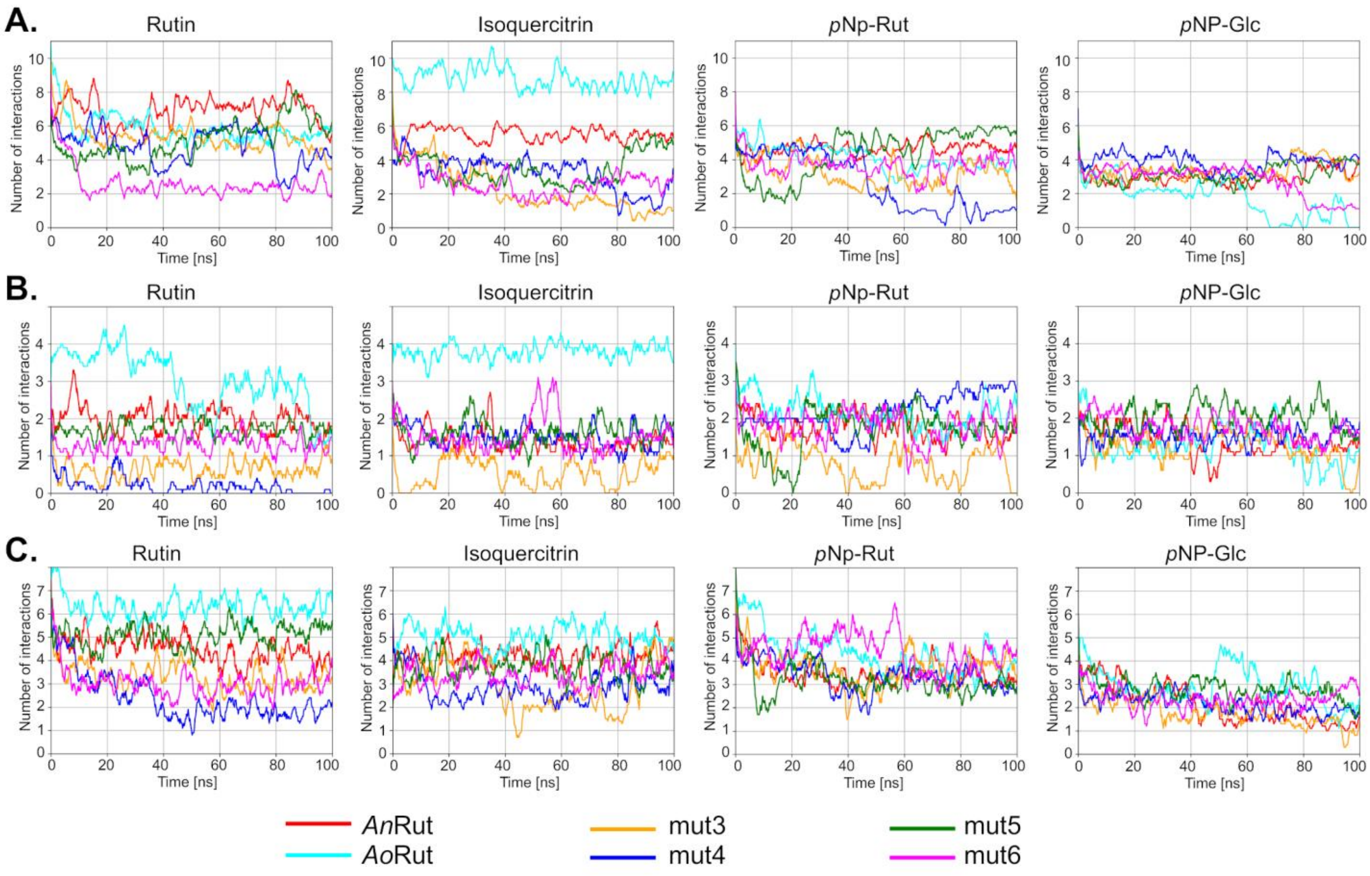

Figure S16. Interactions of substrates with enzymes: **A.** Number of hydrogen bonds formed by substrates with the enzymes during MD simulation. **B.** Number of π-stacking interactions. **C.** Number of hydrophobic interactions.

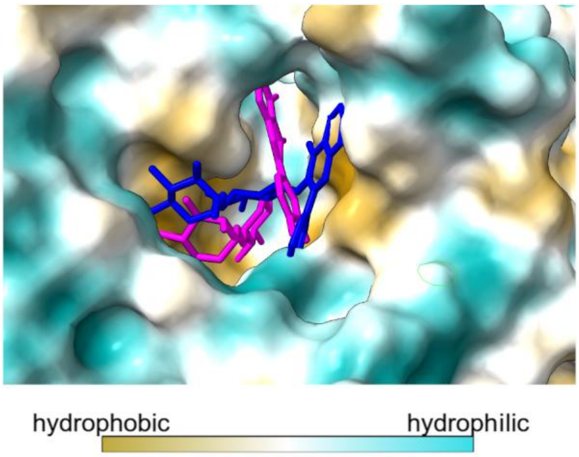

Figure S17. Changed orientation of rutin (**7**) in *An*Rut mut4. Overlay of the position of rutin (**7**) in the active site of *An*RUT WT (magenta) and mut4 (blue) after 100 ns of MD simulation shows reorientation of the quercetin aglycone in mut4. Molecular surface is represented for *An*Rut WT and colored by hydrophobicity.

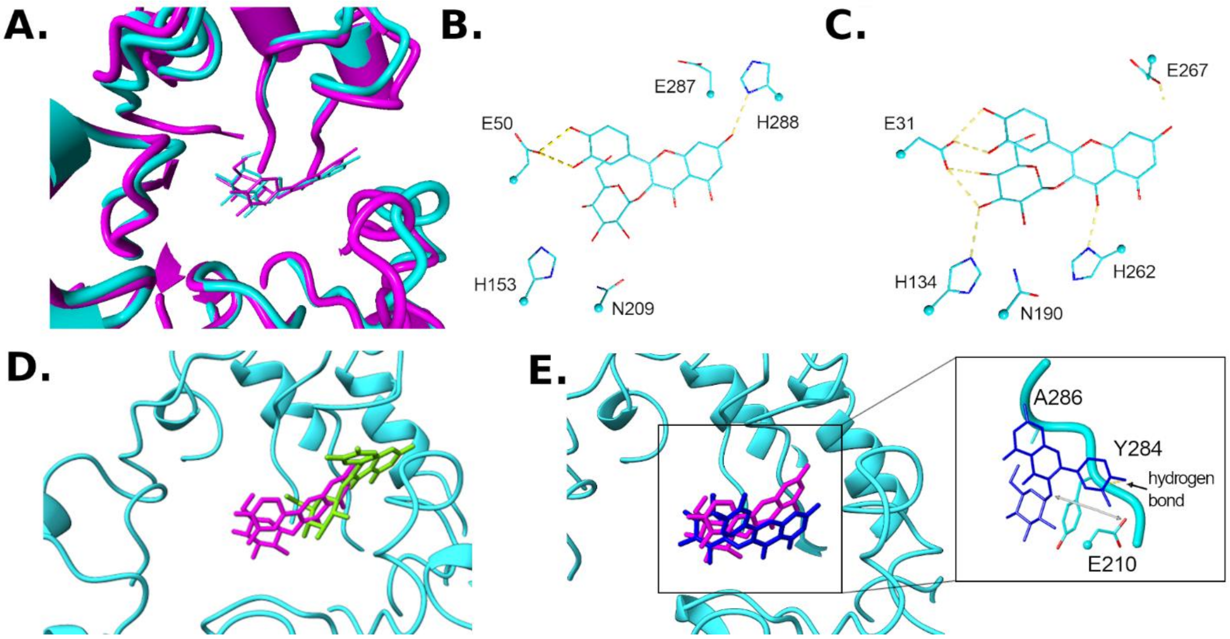

Figure S18. Changed orientation and interactions of isoquercitrin (**8**) in rutinosidase variants. **A.** Structural alignment of *An*Rut WT (magenta) and *Ao*Rut (cyan) with bound isoquercitrin after 100 ns of MD simulation shows a similar position of **8** in the enzyme active sites. **B**. Hydrogen bond interactions of isoquercitrin in the active site of *An*Rut WT after 100 ns of MD simulation. Catalytic residues (E210, E319) are hidden, hydrogen bonds are depicted as yellow dashed lines. **C**. Hydrogen bond interactions of isoquercitrin in the active site of *Ao*Rut WT after 100 ns of MD simulation. Catalytic residues (E191, E298) are hidden, hydrogen bonds are depicted as yellow dashed lines. **D.** Changed position of isoquercitrin in the active site of *An*Rut mut3 (green) overlaid with the position in *An*Rut WT (magenta) after 100 ns of MD simulation; the structure of mut3 is shown in cyan. **E.** Changed position of isoquercitrin in the active site of *An*Rut mut4 (blue) overlaid with the position in *An*Rut WT (magenta) after 100 ns of MD simulation; the structure of mut4 is shown in cyan. The inset shows detailed changes of isoquercitrin position in mut4: increased distance from catalytic residue E210 to 0.65 nm and formation of a new hydrogen bond with Y284, not found in WT. Mutated residue A286 is labeled.

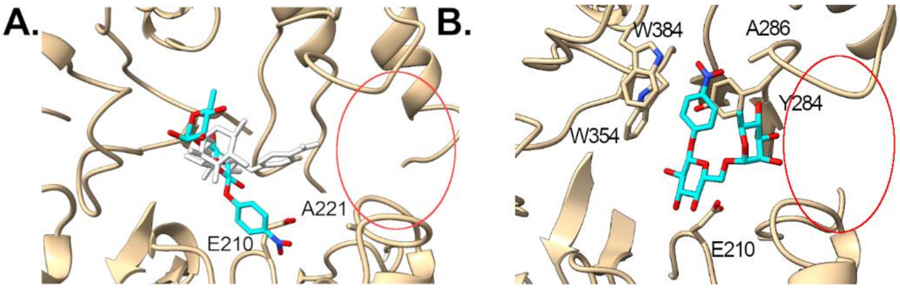

Figure S19. Changes in the position of *p*NP-Rut (**5**) in *An*Rut mut3 and mut4. Hydrogens are hidden. The position of the side tunnel/groove is shown by a red circle. **A.** Changed position of *p*NP-Rut in the active site of mut3 with respect to *An*Rut WT after 100 ns of MD simulation. *p*NP aglycone (element colors: carbon – cyan, oxygen – red , nitrogen – blue) in mut3 (secondary structure, ecru color) occasionally occupied the site freed by the F221A mutation. The distance between the substrate and catalytic E210 in mut3 increased to 0.63 nm. For comparison, the position of *p*NP-Rut in *An*Rut WT is shown in light grey. **B.** Changed position of *p*NP-Rut in the active site of mut4 with respect to *An*Rut WT after 70 ns of MD simulation. *p*NP aglycone occupied the site freed by the Y286A mutation, and formed π-stacking interactions with Y284. The distance between the glycosidic oxygen and the catalytic E210 increased to 0.56 nm.

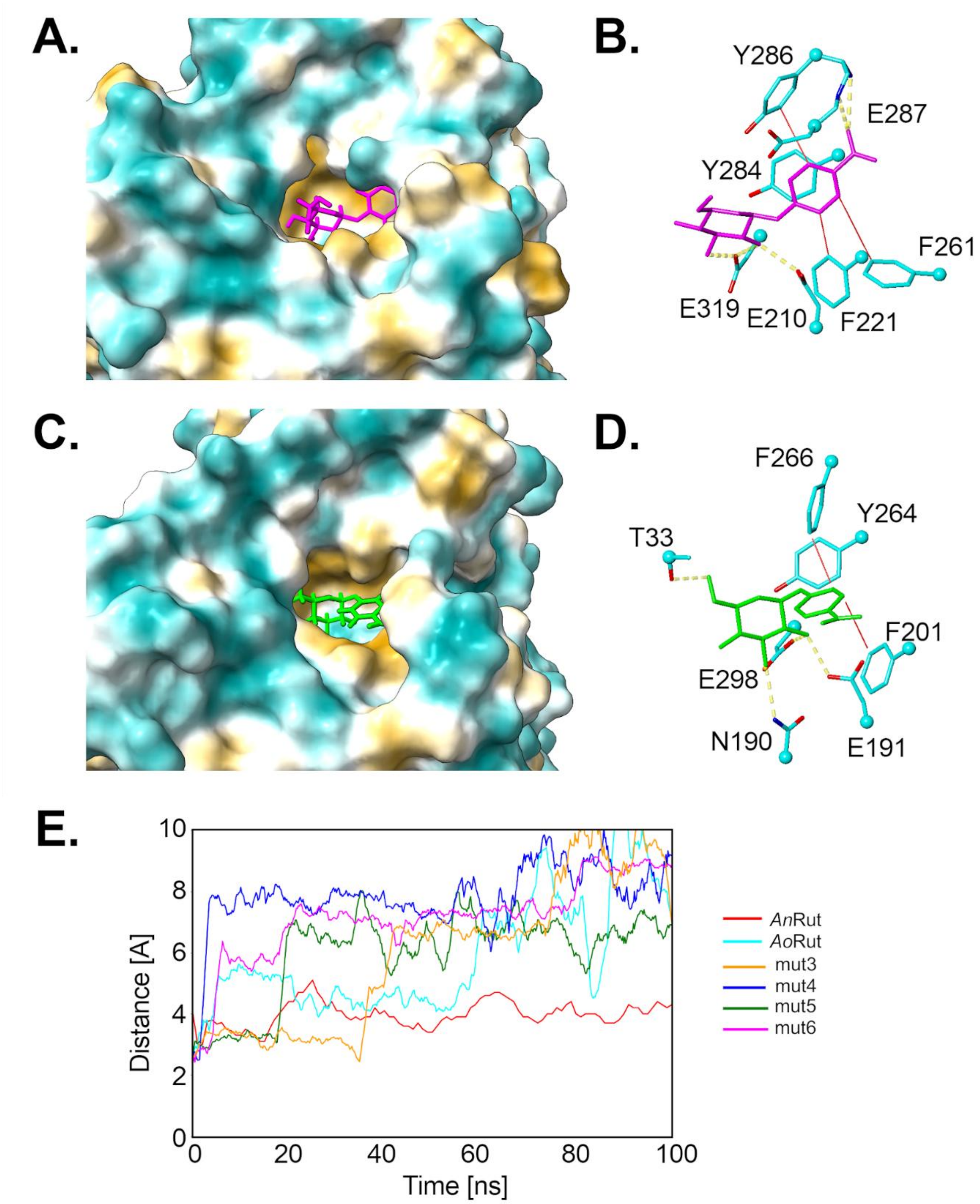

Figure S20. Changes in the position and interactions of *p*NP-Glc (**6**) in rutinosidase variants. π-Stacking interactions are shown by thin red lines, hydrogen bonds by yellow dashed lines. **A.** Position of *p*NP-Glc (magenta) in the active site of *An*Rut WT (hydrophobic surface representation) after 100 ns of MD simulation. **B.** Interactions of *p*NP-Glc in the active site of *An*Rut WT after 100 ns of MD simulation. **C.** Position of *p*NP-Glc (green) in the active site of *Ao*Rut (hydrophobic surface representation) after 79 ns of MD. **D.** Interactions of *p*NP-Glc in the active site of *Ao*Rut after 79 ns of MD simulation. **E.** Distance between the glycosidic oxygen of *p*NP-Glc and the hydrogen of the catalytic residue E210 (for *An*Rut variants) or E191 (for *Ao*Rut) during MD simulation.

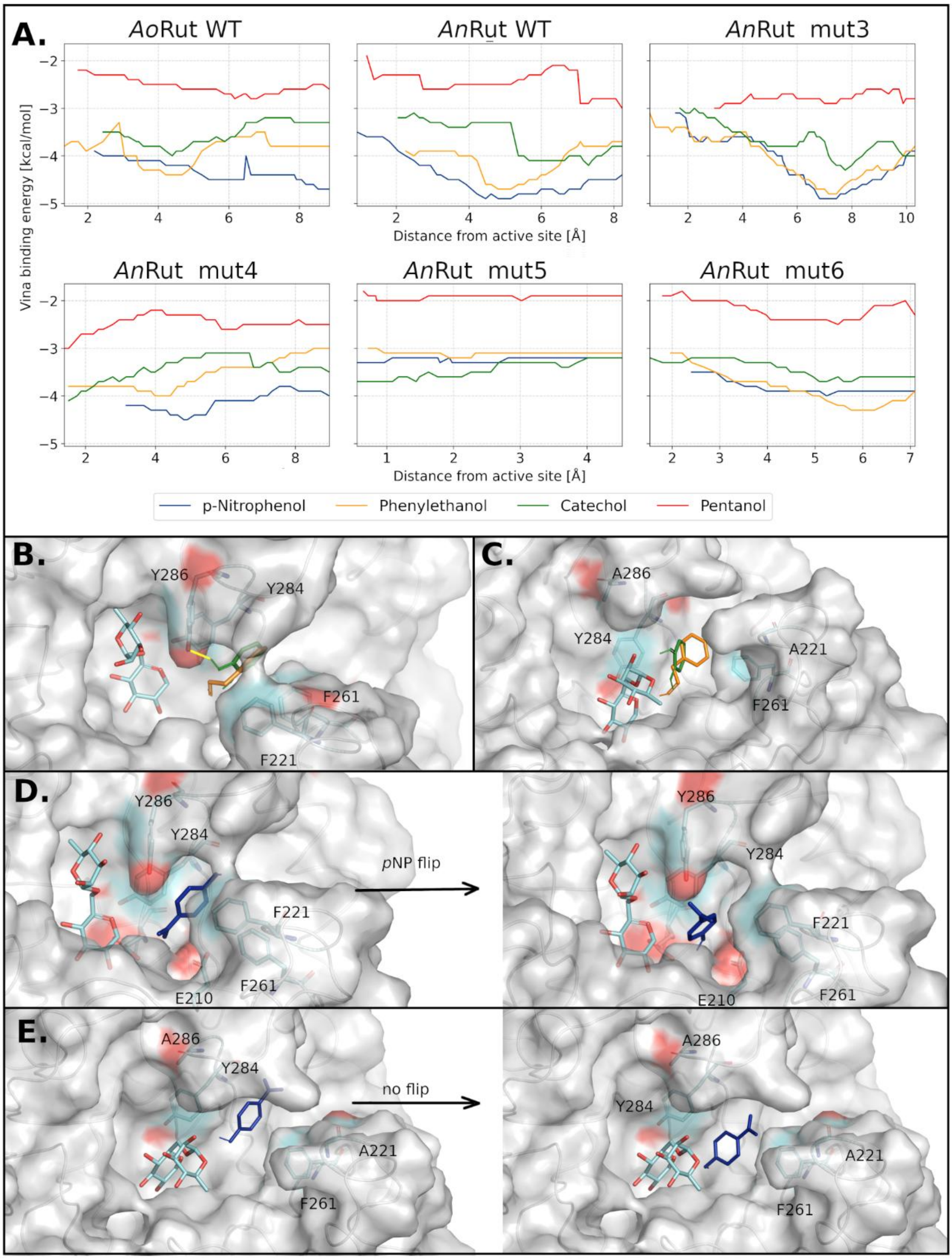

Figure S21. (**A**) The energy profiles of the transglycosylation acceptor pathway from the active site through the side tunnel/along the side groove. The active site was assigned as the starting point with distance zero (0 Å) on the x-axis. The x-axis shows the distance of the passing acceptor from its final position in the active site through the side tunnel/along the side groove. We analyzed the energy difference between the acceptor position at 0 Å (*i.e.*, in the active site) and at the entrance to the tunnel – the so-called 'enthalpy of binding'.[5] If the binding energy at the entrance of the side-tunnel (the furthest point on the x-axis) was lower than in the active site (at 0 Å), we assumed that the acceptor did not have the driving force to go deeper into the tunnel to enter the active site through the side tunnel. Pentanol displayed the highest binding energy to the residues along the tunnel/groove among the tested transglycosylation acceptors, with limited interactions with side-tunnel residues. This behavior is consistent with its relatively low transglycosylation efficiency observed experimentally. In *An*Rut mut3, the energy profile for *p*-nitrophenol and phenylethanol revealed an energy drop at a distance of 7 Å (at the beginning of the tunnel) as a result of hydrogen bonds with residues K307 and D303. Therefore, in mut3, passage of these acceptors through the tunnel

was less energetically favorable. (**B**) For catechol (green) and phenylethanol (orange), a significant energy drop at 4.5–5 Å during the passage through the side tunnel of *An*Rut WT indicated stabilizing interactions with the hydrophobic patch (Y286, F261, F221, Y284) just in front of the active site and hydrogen bond interaction with Y286. These interactions influenced the orientation of the transglycosylation acceptor relative to the transglycosylation donor, but they may have caused the acceptors to become stuck in the tunnel and do not proceed further. The aromatic residues close to the active site, F221, F261, Y286, and Y284, are shown in *An*Rut WT. (**C**) The positions of catechol (green) and phenylethanol (orange) in the groove of *An*Rut mut4 showed unhindered access because hydrophobic interactions are weaker in mutant variants with altered tunnel geometries and a less aromatic core. The π-stacking interaction may have a positive stabilizing effect once the acceptor reaches the active site. (**D**) *p*-Nitrophenol (blue) exhibited difficulties entering the narrow side tunnel in *An*Rut WT in the correct orientation for catalysis. It may flip in the active site to the proper position, but this flip is energetically unfavorable. (**E**) The wider groove of mut4 improves accessibility to the active site, consistent with its superior transglycosylation performance with this acceptor. The rutinosyl transition state intermediate is shown in element colors (cyan for carbon) in panels B–E.

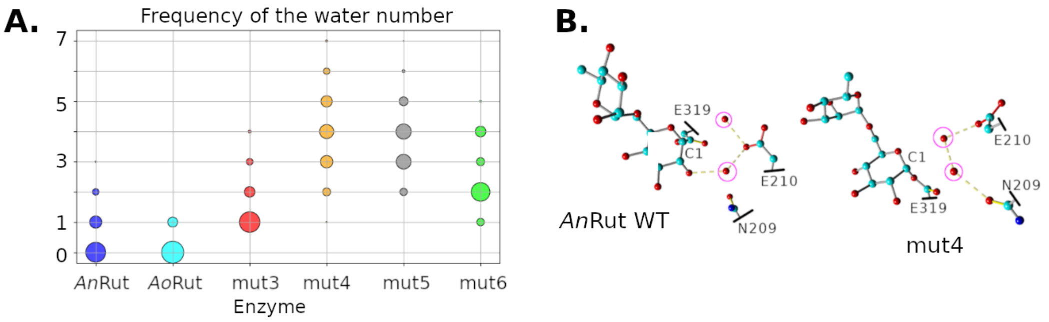

Figure S22. Difference in the number of water molecules close to the catalytic residue and the rutinosyl-enzyme intermediate. A. Frequencies of water molecules transiently resting within 4 Å of the rutinosyl-enzyme intermediate or the catalytic acid/base residue during MD simulations of *An*Rut WT, *Ao*Rut WT and *An*Rut mutant variants during 50-100 ns of MD simulation. **B.** Water molecules within 4 Å of the rutinosyl-enzyme intermediate or the catalytic acid/base residue are highlighted by magenta circles. Hydrogen bonds involving water molecules are shown. Hydrogen atoms are not shown for better clarity.

5. References

(1) Backinowsky, L. V.; Balan, N. F.; Shashkov, A. S.; Kochetkov, N. K. *Carbohydr. Res.* **1980**, *84*, 225-236. DOI: 10.1016/S0008-6215(00)85553-6.

(2) Baumes, R. L.; Bayonove, C. L.; Cordonnier, R. E.; Günata, Y. Z.; Wylde, R.; Heitz, A. *Carbohydr. Res.* **1989**, *189*, 331-340. DOI: 10.1016/0008-6215(89)84109-6.

(3) Šimčíková, D.; Kotik, M.; Weignerová, L.; Halada, P.; Pelantová, H.; Adamcová, K.; Křen, V. *Adv. Synth. Catal.* **2015**, *357*, 107-117. DOI: 10.1002/adsc.201400566.

(4) Koseki, T.; Ichikawa, K.; Sasaki, K.; Shiono, Y. Characterization of a novel *Aspergillus oryzae* tannase expressed in *Pichia pastoris*. *J. Biosci. Bioeng*. **2018**, *126*, 553-558. DOI: 10.1016/j.jbiosc.2018.05.010

(5) Vavra, O.; Filipovic, J.; Plhak, J.; Bednar, D.; Marques, S. M.; Brezovsky, J.; Stourac, J.; Matyska, L.; Damborsky, J. *Bioinformatics* **2019**, *35*, 4986–4993. DOI: 10.1093/bioinformatics/btz386.

6. Abbreviations Used

*An*Rut, rutinosidase from *Aspergillus niger*
*Ao*Rut, rutinosidase from *Aspergillus oryzae*
BMGY, buffered glycerol complex medium
BMMY, buffered methanol complex medium
DMSO, dimethyl sulfoxide
ELSD, light scattering detector
ESI-MS, electrospray ionization mass spectrometry
HPLC, high-performance liquid chromatography
MD, molecular dynamics
MS-ESI, mass spectrometry with electrospray ionization
NPT, isothermal-isobaric ensemble
PDA, photo diode array detector
*p*NP, *p*-nitrophenol
*p*NP-Glc, 4-nitrophenyl β-D-glucopyranoside
*p*NP-Rut, *p*-nitrophenyl rutinoside (4-nitrophenyl 6-*O*-(6-α-L-rhamnosyl)-β-D-glucopyranoside)
RDF, radial distribution function
RMSD, Root mean square deviation
RMSF, Root mean square fluctuation
Rut, rutin
SDS-PAGE, sodium dodecyl sulfate-polyacrylamide gel electrophoresis
TG/H ratio, transglycosylation/hydrolysis ratio
WT, wild type
YPDS, yeast pepton dextrose sorbitol agar
